# Literature Review of the In-Plane Behavior of Masonry Walls: Theoretical vs. Experimental Results

**DOI:** 10.3390/ma14113063

**Published:** 2021-06-03

**Authors:** Thomas Celano, Luca Umberto Argiento, Francesca Ceroni, Claudia Casapulla

**Affiliations:** 1 Department of Engineering, University of Naples Parthenope, Centro Direzionale Is. C4, 80143 Napoli, Italy; thomas.celano001@studenti.uniparthenope.it; 2 Department of Structure for Engineering and Architecture, University of Naples Federico II, Via Forno Vecchio, 80134 Napoli, Italy; lucaumberto.argiento@unina.it (L.U.A.); casacla@unina.it (C.C.)

**Keywords:** regular masonry, irregular/rubble masonry, walls, in-plane behavior, shear failures, flexural failures, shear-compression test, design formulations

## Abstract

In-plane strength of masonry walls is affected by the resistant mechanisms activated in the walls, i.e., related to flexural or shear behavior. The latter one can occur in the walls according to different failure modes depending on both mortar and unit strengths and on the type of assembling, i.e., ‘regular’ or ‘irregular’ texture. In this paper, a critical review of the existing design formulations for the in-plane strength of masonry walls is firstly presented, with important information on the achievable failure modes depending on the geometrical and mechanical features of the masonry fabric. Then, experimental tests are collected from the literature and a comparison between theoretical and experimental results is carried out. The presented analyses are aimed to highlight the differences between the existing formulations and to identify the most suitable ones.

## 1. Introduction

Under dynamic actions (i.e., earthquakes, wind, etc.), masonry buildings can collapse or have relevant damage for two main groups of mechanisms: ‘local’ and ‘global’ failure modes. The loss of stability of parts of the structure determines local failure mechanisms [1,2,3,4,5], while all the crises related to the in-plane behavior of masonry walls can be addressed to the global ones. Focusing on the second group, the failure mechanisms are called ‘global’ because they can occur only if the whole structure reacts as a box, according to the so-called ‘box-behavior’. In this case, efficient connections among walls and between walls and floors are able to prevent out-of-plane failures [6,7] and in-plane mechanisms can take place [8].

**Figure 1:**
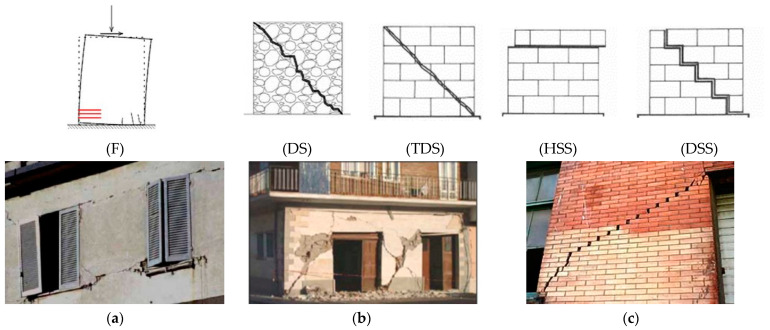
Typical failure mode in masonry panels: (**a**) flexural failure (F); (**b**) diagonal shear failure mode (DS or TDS); (**c**) sliding shear failure (HSS or DSS).

The ‘in-plane’ failure modes are generally categorized as follows (see Figure 1):

- flexural failure (rocking or toe crushing, named F in Figure 1a): failure due to the achievement of the tensile or compressive strength along the cross end-sections of the wall and characterized by nearly horizontal or vertical cracks, respectively;
- diagonal shear failure (Figure 1b): failure related to the achievement of the tensile strength of masonry along the principal direction and characterized by diagonal cracks along the wall. This kind of failure generally occurs in irregular/rubble masonry (DS), or in regular masonry (TDS) with strong mortar/weak units and good bond behavior at the mortar-masonry interfaces;
- sliding shear failure (Figure 1c): failure occurs along the mortar joints according to horizontal (HSS) or stepped diagonal cracks (DSS) because of the low bond strength at the mortar-masonry interface or in case of reduced values of the compressive stresses acting in the wall.

Several authors have studied the influence of different parameters on the in-plane capacity of masonry walls [9,10,11,12,13,14,15,16,17,18,19,20,21]. Among these, the following five main parameters can be identified: (a) mechanical properties of the material, (b) constraint conditions, (c) vertical compression level, (d) slenderness of the wall, (e) masonry texture.

The dependence of the in-plane failure on the mechanical properties of the material is clear: all failure modes are related to the achievement of the local strength of the material and, in particular, of the tensile strength. The constraint conditions and the slenderness of walls are also very important aspects because they may favor the formation of diagonal or horizontal cracks and, thus, drive the failure mechanisms (shear or flexural failure). The vertical compression level influences both the capacity and the type of failure: for a low state of compression, a sliding shear failure is, indeed, generally favored in regular walls. For moderate and high compression levels, flexural and diagonal shear failures may occur depending on other parameters (slenderness and constraints). The in-plane slenderness, generally defined as the height-to-width ratio of the wall, influences the distribution of the normal and tangential stresses inside the wall. For slenderness equal to or lower than 1 (so-called ‘squat’ walls), the shear mechanisms are, indeed, predominant in comparison with the flexural one, which is mainly exhibited by walls with slenderness more than 1.5 (so-called ‘slender’ walls).

Finally, the masonry texture also has a relevant role on the most probable failure mode of walls: clearly, sliding shear failures, as evidenced in Figure 1c, can occur only when regular mortar joints are present, i.e., ‘regular’ masonry. In the case of chaotic masonry, i.e., ‘irregular/rubble’ masonry, indeed, the geometrical interlocking between masonry units and/or elements with several dimensions allows to avoid sliding phenomena and leads to diagonal shear failures. It is important to note that the anisotropic behavior of the masonry material is strongly dependent on the ratio between the strength/stiffness of units and mortar joints [22].

From the experimental point of view, two types of tests have been diffusely used by researchers to investigate the in-plane behavior of masonry walls under lateral loads. These are:

- diagonal compression test;
- shear-compression test.

Diagonal compression tests are mainly used to determine the tensile strength of masonry [23,24], considering the walls subject to the only compression force applied along a diagonal in the absence of lateral compression, in order to determine a pure shear stress condition in the middle of the wall.

Shear-compression tests allow to better analyze the effective in-plane response of masonry walls because a biaxial stress state, due to the simultaneous application of vertical compressive and horizontal shear forces, takes place [11,12,14,25,26,27,28,29,30,31,32,33,34,35,36]. Such a loading condition more reliably represents the behavior of walls inserted in masonry structures under vertical and horizontal actions.

In this framework, there is a wealth of recent literature that has analyzed the in-plane behavior of masonry walls through laboratory or in-situ tests based on these two set-ups, also considering the presence of externally bonded innovative reinforcing systems [37,38,39,40,41,42,43,44,45,46,47,48,49].

The first part of this paper is focused on a detailed analysis of the theoretical formulations available in the literature proposed by several authors and codes to predict the in-plane capacity of masonry walls, with reference to both flexural and shear failure. The existing strength models are classified according to the failure modes achievable in regular and irregular masonry walls. An accurate sensitivity analysis of the formulations to the parameters involved is reported as well, in order to assess the differences between them and identify the safest ones. Successively, a database of experimental tests (i.e., shear-compression tests) on masonry walls available in the literature and made of both regular and irregular/rubble masonry is collected in order to assess the reliability of the previously examined formulations. Finally, due to their higher easiness of application, it was also explored if the formulations usually provided for the shear strength of irregular/rubble masonry walls are safe for predicting the shear strength of regular masonry walls, too.

## 2. Literature Review of Theoretical Formulations

Several formulations are available in the literature about the in-plane capacity of masonry walls, referenced to each of the failure mechanisms described above. In order to numerically evaluate this capacity, the available formulations referred to the cases of flexural and shear failures shown in Figure 1 are briefly described in the following. These will then be used for comparisons with available experimental results on masonry walls (Section 4).

### 2.1. Flexural Failure

**Figure 2:**
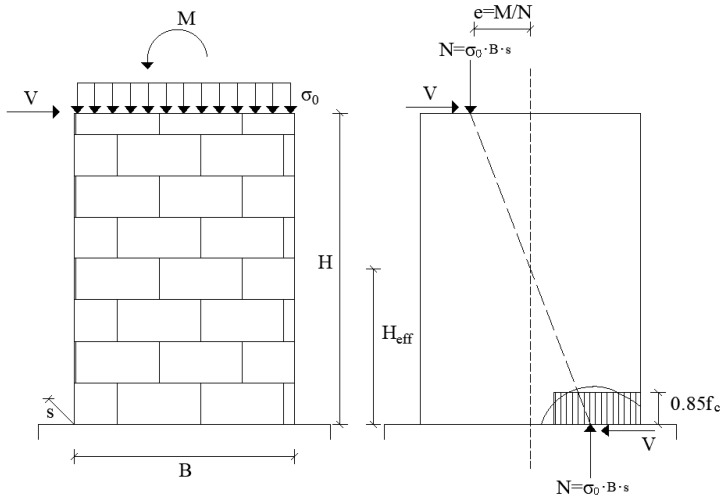
Equilibrium of masonry walls in case of flexural failure.

The most ductile mechanism is attained in the case of flexural failure, which is generally studied considering a certain distribution of the normal stresses at the base of the wall subjected to both horizontal (shear force) and vertical (normal forces corresponding to gravitational forces) loads. The flexural capacity is directly derived by the global and the local equilibrium of the wall and of the cross end-sections. Depending on the distribution of the normal stresses at the base of the wall and on the constraint conditions, different authors have proposed various formulations for the flexural strength, usually expressed in terms of maximum shear force (Table 1). The formulations reported in the following refer to masonry walls having base width *B*, height *H*, and thickness *s*, subject to an average normal stress *σ*_0_, due to the vertical force *N*, applied with eccentricity *e* = *M/N*, and to a horizontal force representative of the shear action, *V* (Figure 2).

It is worth noting that the self-weight of the wall is generally neglected because it is very low with respect to the average normal stress, *σ*_0_, applied during the experimental tests.

Tomaževič and Lutman [50] have provided a theoretical formulation to predict the flexural capacity of masonry walls, i.e., Equation (1) in Table 1. It was obtained through an internal and external equilibrium analysis of a masonry wall, with in-plane slenderness *λ* = *H*/*B* and a double fixed constraint condition at the ends represented by the parameter *ψ*. The theoretical results were compared with the outcomes of several experimental tests performed by the same authors [12], evidencing a good agreement.

Magenes and Calvi [42] have adopted the same approach of [50] with a slight difference in the compressive strength, providing Equation (2) in Table 1. These authors have suggested using such a formulation when the value of the aspect ratio, *ψλ*, is higher than 1. In the work of Abrams [51], the formulation used to predict the flexural capacity of masonry walls, i.e., Equation (3) in Table 1, is based on the FEMA-273 guidelines. Still, this formulation is closely comparable with the previous two, considering a further reduced compressive strength.

Both Eurocode 8-Part 3 [52] and Italian building code [53] provide very similar formulations for predicting the flexural capacity of masonry walls, represented by Equations (4) and (5) in Table 1, respectively.

It should be noted that all the formulations listed in Table 1 have the same structure since they are obtained following the same rotational equilibrium analysis of the wall according to a double-fixed boundary condition. In order to account for different constraint conditions at the ends, in some formulations, the shear length, *H_eff_*, is defined as the distance between the cross end-section of the wall and the no-bending moment section, as shown in Figure 2. The distribution of compressive stresses on the base wall section towards the edge is idealized as constant (stress-block simplification) by means of a reduction factor, in order to facilitate the analysis.

In Table 1, the values of the compressive strength and the aspect ratio adopted in the various formulations are listed too. The aspect ratio, *ψλ*, depends on the wall dimensions (*H* and *B*), and on the constraint conditions through the parameter *ψ*. As reported in Table 1, this parameter is 1 in a cantilever scheme and 0.5 in a double-fixed constrain condition. In Equations (1) and (2), *ψ* is directly indicated, while for Equations (3)–(5) it is considered through *H_eff_*, which is equal to H/2 in a double-fixed condition and to *H* for the cantilever.

Moreover, the flexural capacity depends on the compressive stress state at the cross end-section of the wall, *σ*_0_, and on the compressive strength, *f_c_*, of masonry. About the latter, in Equations (2) and (3) the average strength, *f_c_*_,*av*_, of masonry is assumed, while in the others the design value, *f_c_*_,*d*_, is used. It is worth noting that the design values are calculated differently for new and existing masonry buildings, according to the indications of the Commentary to the Italian code [54]. While the formulation reported in Eurocode 6 [55] is focused on new masonry buildings, Eurocode 8-Part 3 [52] provides a slightly different formulation for existing masonry buildings. Similar to what is indicated in [54], for existing buildings the use of mean values for strengths and of ‘confidence’ factors, depending on the knowledge level attained in the structure, is suggested.

About the normal compressive stress distribution, in Equation (1) the whole value of the compressive strength is assumed, while in Equations (2), (4) and (5) a 15% reduction is adopted, and in Equation (3) the reduction becomes 30%. These reduction factors are related to the assumption of a stress-block distribution that facilitates the analysis of the section by assuming a constant distribution of the normal compressive stresses instead of the real one related to the constitutive law in compression of masonry (Figure 2). However, it is easy to derive that the shear capacity of the wall in case of flexural collapse is much more influenced by the aspect ratio, *ψλ*, rather than by the strength reduction, and the choice of the constitutive law in compression for masonry does not influence significantly the prediction of the shear capacity [42].

### 2.2. Shear Failure

Shear failure is, in general, a brittle mechanism in comparison with the flexural one, with a certain ductility attained in the case of sliding mechanisms along the mortar joints. Shear failure is accompanied by the propagation of oriented cracks and mainly occurs for relatively high axial loads and squat elements, i.e., in elements where bending stresses are lower. The cracks can extend either through the mortar or the units, depending on their strengths and on the anisotropy of the material [13].

As introduced above, two main shear failures can be considered: (a) sliding shear failure, (HSS or DSS) and (b) tension diagonal shear failure (TDS or DS). The sliding shear failure is typical of masonry with regular texture subjected to low-medium axial loads, while the second one can occur both in regular and in irregular/rubble masonry textures under greater axial loads. A description of these typical shear failures is presented in the following sections, based on the type of masonry, i.e., regular or irregular/rubble masonry.

#### 2.2.1. Regular Masonry

**Figure 3:**
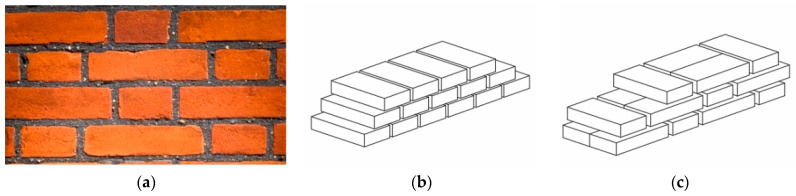
(**a**) Example of regular masonry texture; (**b**) texture with units in the same direction; (**c**) texture with units in two directions.

‘Regular’ masonries are characterized by a regular disposition of block/brick with or without mortar joints, as shown in Figure 3a. Figure 3b,c show two possible examples of textures for regular masonry walls along with the thickness: the presence of transversal units (Figure 3c) may provide a monolithic behaviour to the whole wall and, thus, it is an important requirement for activating its in-plane behavior.

For walls made of regular masonry, the two in-plane shear failures are: (a) sliding shear failure (HSS or DSS in Figure 1c), and (b) diagonal shear failure with cracking of units (TDS in Figure 1b). Thus, these two shear failure modes are governed by the cohesion of joints and the tensile strength of units, respectively. Usually, sliding shear failure (HSS or DSS) is related to the crisis of the bond at the mortar-masonry interface and is the predominant failure mode if the ratio between the shear strengths of units and mortar is higher than 1 (strong units and weak mortar) and when the compression level is limited. In the case of weak mortar, indeed, cohesion is low and sliding failure can occur. Such a sliding mechanism is commonly studied through the application of the well-known Mohr–Coulomb criterion, which provides the shear strength, *τ*, based on cohesion, *c*, and the friction angle, *φ*, of the material, and the level of normal compression, *σ*, applied in the wall:

*τ* = *c* + tan *φ* ∙ *σ* (6)

Equation (6) shows that the level of vertical compression clearly plays a fundamental role since it increases the frictional contribution. It is worth noting that, in the formulations listed in Table 2, the friction coefficient, *μ* = tan *φ*, is directly adopted, the average normal stress, *σ*_0_, is considered, and the cohesion, *c*, is usually indicated as *f_v_*_0_, i.e., the shear strength in the absence of normal stresses. In particular, *c* and *φ* are mechanical parameters generally assessed by means of bond tests on two (couplet) or three (triplet) masonry units connected by mortar [56].

On the basis of experimental tests carried out on masonry walls, Grimm [57] has proposed a formulation (Equation (7) in Table 2) dependent on the parameters *f_v0_* and *µ*, defined as ‘local’ values of cohesion and friction coefficient, respectively, and on the length of the uncracked part of the cross end-section of the wall, defined as *B*’.

Generally, local parameters are used to predict the shear capacity of masonry walls that exhibit a sliding shear failure along the adjacent bed joints (i.e., HSS failure mode in Figure 1c). This is an important aspect since local parameters can be experimentally obtained by means of simple tests, such as couplet or triplet tests, and are related to the local adherence developed at the interface between units and mortar. Note that in Equation (7) local cohesion is increased by 40% in order to take into account the interlocking phenomenon.

Eurocode 6 [55] and the Italian building code [53] provide a similar formulation based on Equation (6) (i.e., Equation (8) in Table 2), without increasing the local cohesion, but using the design coefficient, *γ_d_*. According to the Italian code, this formulation is generally used for new buildings, while more detailed indications for existing buildings are reported in the Commentary [54] and are represented by Equations (11) and (12) in Table 2.

Conversely, other literature formulations use the global parameters *f_v0’_* and *µ’* of masonry, which are referred to the global behavior of masonry walls accounting for the interlocking effect among the units and the full length of the cross section, *B*. The global parameters are obtained by modifying the local parameters, *f_v0_* and *µ*, through the unit shape ratio, *φ =* 2*h_b_/b_b_*, where *h_b_* and *b_b_* are the height and the width of the single unit, respectively. It is important to highlight that, while local parameters are used to define the shear sliding failure along a horizontal crack (HSS), global parameters are mainly used to predict the shear capacity in the case of diagonal sliding shear failure (DSS), i.e., in the case of sliding along diagonal stepped cracks.

**Figure 4:**
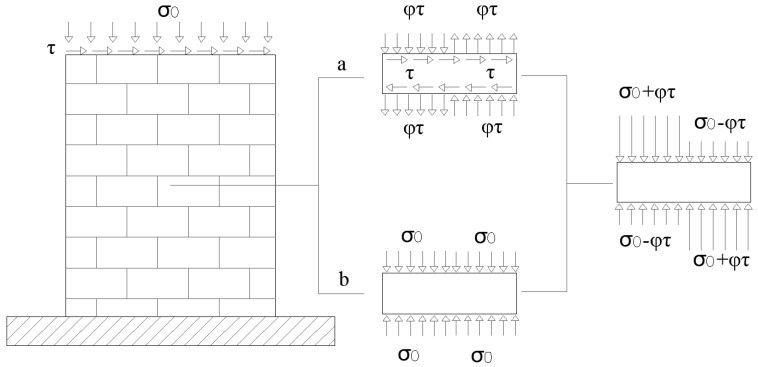
Distribution of the normal stress according to Mann and Muller (From Calderini et al. [22]).

In order to predict the strength associated to the DSS failure, the Mohr–Coulomb criterion, i.e., Equation (6), is applied with reference to a local state of damage in the middle cross section of the masonry wall (Figure 4). Such an approach is followed by Mann and Muller [58], who have proposed a formulation (Equation (9) in Table 2) based on the equilibrium equations of a single unit and on two main hypotheses: (1) head joints have negligible mechanical properties (no normal and tangential stresses) and (2) the ratio between the stiffness of units and mortar is very high (rigid units). According to this model, the compressive normal stresses on a single unit in the middle cross section can be evaluated as a sum of the stresses produced by vertical loads (stress distribution ‘b’ in Figure 4) and the ones related to shear actions (stress distribution ‘a’ in Figure 4). In fact, because no stresses are assumed along the head joints, the rotational equilibrium of the unit in the latter distribution can only be satisfied by a variation of the normal stresses along the bed joints, where *φ* is the unit shape ratio.

Magenes and Calvi [42] have also proposed a formulation (Equation (10) in Table 2) for assessing the shear strength of walls made of regular masonry using the global parameters and, thus, with reference to the DSS failure in Figure 1c. Similar to what was proposed by Mann and Muller [58], Equation (10) defines the global parameters *f_v0_*’ and *µ*’, through the unit shape ratio, *φ*. However, with respect to the formulation of Mann and Muller [58], the slenderness of the wall, *λ*, is introduced to take into account the effect of stress distributions, the interaction between shear and flexural stresses, and the crack propagation, while the parameter *ψ* is always used to account for the effect of the constraint conditions. The authors have compared the theoretical values of the capacity provided by the proposed formulation with the results of quasi-static cyclic experimental tests, performed by Anthoine et al. [11] on clay brick masonry walls with different slenderness and a double-fixed constraint condition. A significant scatter between theoretical and experimental capacities was observed, mainly related to the scatter of the mechanical properties assumed for masonry.

The Commentary to the Italian code [54] provides a difference for walls made of regular and irregular/rubble masonries and uses a variety of values of material strengths for new and existing buildings. In particular, for regular masonry, in addition to the HSS failure predicted by Equation (8), the same formulation proposed by Mann and Muller [58] and based on global parameters (Equation (11) in Table 2) is provided for predicting the shear strength in the case of sliding along diagonal stepped cracks in the mortar (DSS failure). However, the document also provides an upper bound (Equation (12) in Table 2) for the shear strength of walls made of regular masonry, which is represented by the shear-tensile cracking of units, *V_t_*_,*lim*_, depending on their tensile strength, *f_bt_*_,*d*_.

It is worth noting that Equations (9) and (11) contain an additional parameter, *b*, called ‘shape factor’, which takes into account the shear stress distribution along the middle cross section of the wall by means of the maximum-to-average shear stress ratio. The shear distribution is influenced by the geometry of the wall as some numerical analyses have evidenced [13,59,60] and in many formulations the shape factor is generally defined as 1 ≤ *b* = *λ* = *H/B* ≤ 1.5, i.e., it is assumed equal to the slenderness of the wall, *λ*, but limited to the range 1–1.5. Such a definition is based on the assumption that the distribution of the shear stresses in the middle cross section of the wall is parabolic or constant for very slender or very squat walls, respectively, and this corresponds to have a maximum-to-average shear stress ratio equal to 1.5 or 1 as threshold values. Since the classification of slender or squat wall depends on the slenderness ratio, *λ*, it is more generally assumed that *b* = *λ* in the range 1–1.5. However, such an assumption is not always realistic. Thanks to the analysis of the internal stresses inside a regular masonry wall by means of finite element (FE) models, Betti et al. [59] have proposed, indeed, to assume *b* = (1 + 0.5*λ*) ≤ 1.5, evidencing that the values provided by Equations (9) and (11) are not realistic for squat walls, i.e., *H*/*B* = 1, since they tend to overestimate the effective DSS strength. Moreover, also in Celano et al. [60], numerical analyses with a FE model evidenced that the assumption of parabolic distribution of the shear stresses is reliable even for squat walls.

#### 2.2.2. Irregular/Rubble Masonry

**Figure 5:**
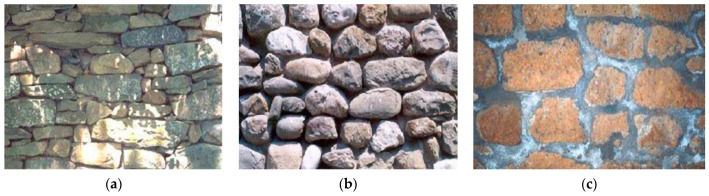
Examples of irregular/rubble masonry: (**a**) semi-finished stone masonry; (**b**) stone masonry with rounded ashlar; (**c**) irregular tuff masonry.

Irregular/rubble masonry walls are made of chaotic arrangements of units with mortar not organized in regular layers such as, e.g., the three different types of stone masonry walls shown in Figure 5. An almost negligible presence of mortar layers can be observed in Figure 5a,b, while the masonry wall in Figure 5c is made of irregular tuff stones bonded with high thickness mortar joints.

In general, a non-regular configuration influences the in-plane behavior of this kind of masonry with reference to both vertical and horizontal actions. In terms of shear failure, irregular/rubble masonry walls can only exhibit a diagonal shear failure (DS in Figure 1b), with diagonally orientated crack patterns when the tension strength is overcome. Depending on the quality of masonry units and mortar, these cracks may either pass through the units or partly follow the joints and partly pass through the units.

Experimental tests were carried out by several authors [27,61,62,63] for investigating the in-plane shear behavior of irregular/rubble masonry walls. The tests mainly evidenced that the DS capacity of the walls can be evaluated assuming masonry as a homogeneous and isotropic material. One of the most popular is, indeed, the formulation proposed by Turnšek and Čačovič [14], i.e., Equation (13) in Table 3, which represents the reference approach for the other ones. This formulation is based on the analysis of the internal stress state of the wall subjected to the DS failure, considering masonry an elastic, homogeneous and isotropic material that fails when its tensile strength is attained.

The formulation of Tomaževič and Lutman [50] (Equation (14) in Table 3) is similar to the previous one but assumes a reduction of 10% of the shear capacity in order to take into account the effect of cyclic loads. The analytical results obtained with this formulation have also been compared with experimental results by the same authors. A similar approach has also been adopted by Abrams [51] and the Commentary to the Italian code [54], by means of Equations (15) and (16) listed in Table 3, respectively.

All these formulations assume the same shape factor, *b*, adopted for regular masonry walls. This factor is variable between 1 and 1.5 for squat and slender walls, respectively, except for Abram’s formula (Equation (15)), where the slenderness of the wall is directly assumed through the aspect ratio, *ψλ*, also accounting for the constraint conditions of the wall. Moreover, it should be remarked that all the formulations in Table 3 report the design value of the tensile strength of masonry, *f_t_*_,*d*_, except for Equation (13) of Turnšek and Čačovič [14], where the average value of the tensile strength, *f_t_*_,*av*_, is assumed. Thus, the main parameters that influence the DS capacity of irregular/rubble masonry walls are the tensile strength of masonry, *f_t_*, the shape factor, *b*, or the aspect ratio, *ψλ*, and the normal compressive stress, *σ*_0_. The tensile strength of masonry *f_t_* is usually obtained by diagonal compression tests.

## 3. Sensitivity Analysis of the Theoretical Formulations

A sensitivity analysis is herein performed to compare the trend of the flexural and shear strength models proposed by the various authors as the main parameters vary. The mechanical properties of masonry are assumed to vary in the ranges of values reported in the Commentary to the Italian code [54] for existing buildings. In particular, the starting values of the tensile and the compressive strengths are *f_t_* = 0.5 MPa and *f_c_* = 5 MPa, respectively, while the cohesion is *f_v_*_0_ = 0.25 MPa; the friction coefficient is assumed *μ* = 0.58, corresponding to a friction angle of 30°, which is commonly suggested in the lack of further data [54]. These values may be considered representative of a regular masonry made of clay bricks with unit dimensions *h_b_* = *b_b_* = 0.15 m, *s_b_* = 0.25 m and joints made of lime-based mortar. It is worth noting that in the following analyses no differences between average and design values of material strengths are assumed. The starting values of geometrical parameters are *B* = 1 m, *H* = 1 m, corresponding to the in-plane slenderness *λ* = *H/B* = 1, and thickness *s* = *s_b_* = 0.25 m. The normal stress is *σ*_0_ = 0.6 MPa and a double-fixed constraint condition is assumed. Table 4 reports the values of the mechanical and geometrical properties assumed for the reference case.

### 3.1. Flexural Failure

**Figure 6:**
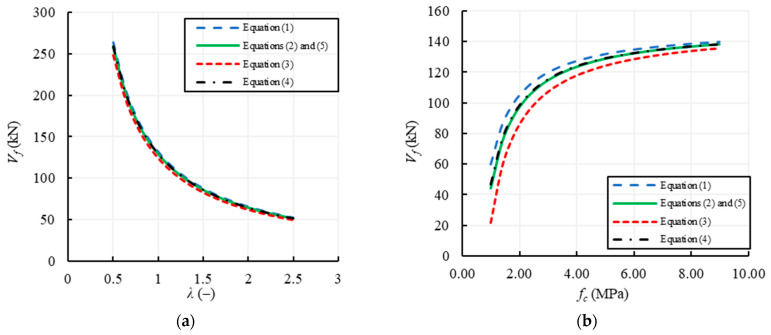
Sensitivity analysis of the flexural capacity provided by several formulations in the function of the: (**a**) wall slenderness, *λ*; (**b**) masonry compressive strength, *f_c_*.

For all the formulations displayed in Table 1, the parameters involved in the evaluation of the flexural capacity of masonry walls are: the geometrical parameters of the wall, i.e., *B*, *H*, *λ* and *s*, the vertical compressive stress, *σ*_0_, the compressive strength of masonry, *f_c_*, and the constraint conditions, evaluated through the factor *ψ*. Among these, the most influencing parameters are *λ* and *f_c_*, which are varied separately in Figure 6, assuming all the other data listed in Table 4 unvaried. In particular, the wall base is assumed constant, *B* = 1 m, while *H* variable from 0.5 m to 2.5 m in order to vary *λ* from 0.5 to 2.5. The compressive strength varies between 1 and 9 MPa, being these values the minimum and the maximum compressive strengths reported in [54]. It is important to underline that, because no difference is assumed between the average and the design value of the compressive strength, Equations (2) and (5) provide the same value of the capacity.

Figure 6a shows how the flexural capacity tends to decrease as the slenderness of the masonry wall increases. Clearly, the curves are almost all overlapping since all formulations depend on the wall slenderness in the same way and there is only some difference in the reduction factor of the compressive strength. Such a decreasing trend of the strength with the slenderness is due to the effect of an increase in the shear length, *H_eff_* (see Figure 2).

Conversely, Figure 6b shows the variability of the flexural capacity with the compressive strength of masonry. In this case, the trend is initially very steep until about *f_c_* ≈ 3 MPa, i.e., until the vertical compression, *σ*_0_, reduces to only 20% of the compressive strength. Then, the capacity increases with the compressive strength up to an asymptotic value and the influence of *σ*_0_ is clearly dampened since it becomes a very low percentage of the compressive strength. Anyway, there is again a slight variability among the results provided by the different formulations, evidencing that normal stresses do not significantly influence the results, especially for large values of *f_c_*. In this sense, the formulation proposed by Abrams [51], i.e., Equation (3), provides the lowest curve since it assumes a higher reduction factor for *f_c_* (i.e., −30%) and the difference with the other formulations is as more relevant as *f_c_* is lower.

### 3.2. Shear Failure of Regular Masonry

The parameters involved in the evaluation of the shear capacity of regular masonry walls due to sliding failure along horizontal cracks (HSS) are: the geometric parameters, *B* and *s*, the reduced length of the section, *B*’, that is the uncracked part of the section on which the sliding occurs (in the specific case, assumed to be equal to *B*/2), the compressive stress, *σ*_0_, the local values of cohesion and internal friction coefficient, *f_v0_* and *μ*. For the formulations using the global parameters, i.e., those aimed to predict the sliding failure along diagonal stepped cracks (DSS), the global values of cohesion and internal friction coefficient, *f’_v0_* and *μ*’, have to be defined in function of the unit shape ratio, *φ* = 2*h_b_/b_b_*, being *h_b_* and *b_b_* the unit dimensions. Note that Equation (10) also depends on the aspect ratio, *ψλ*, while Equations (9) and (11) depend on the shape factor, *b*. Again, due to the assumption of the same value for the average and the design value of the shear strengths, Equations (9) and (11) provide the same shear capacity. It is worth noting that Equation (12) is not plotted here because it is the only one proposed to predict the diagonal shear failure for tensile cracking of units (TDS), and therefore not directly comparable to the others.

For all the examined formulations, the variable parameters assumed in the analyses are *f_v0_*, *μ* and the ratio between the height and length of the unit, *h_b_/b_b_*, which also allows to obtain the global values of cohesion and friction coefficient. In particular, *f_v0_* is varied between 0 and 0.6 MPa, *μ* between 0.1 and 0.80, and *h_b_/b_b_* in 0.1–1.0, the latter range obtained by fixing *b_b_* and changing *h_b_*.

**Figure 7:**
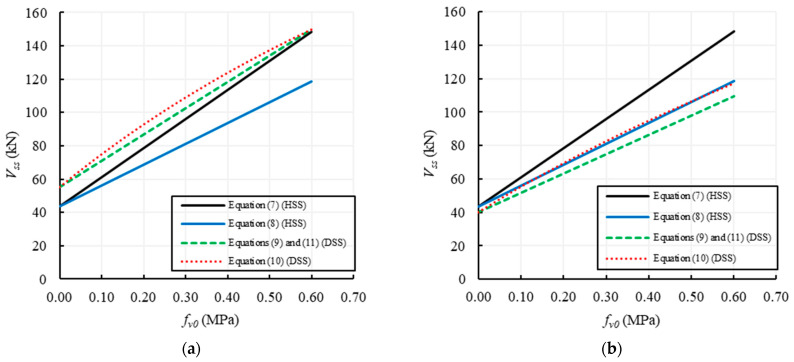
Sensitivity analysis for the shear capacity in regular masonry in function of *f_v0_*, for *µ* = 0.58 and: (**a**) *h_b_/b_b_* = 0.5; (**b**) *h_b_/b_b_* = 1.

**Figure 8:**
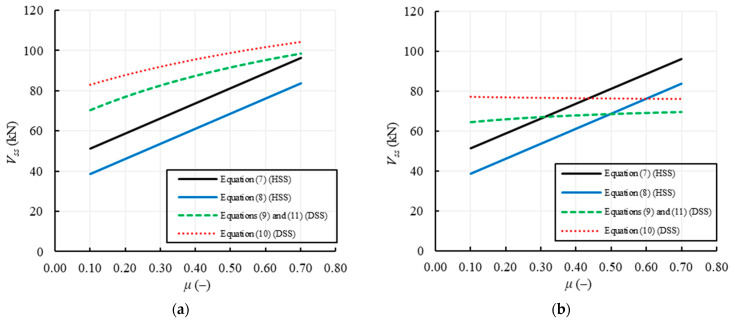
Sensitivity analysis for the shear capacity in regular masonry in function of *μ*, for *f_v0_* = 0.25 MPa and: (**a**) *h_b_/b_b_* = 0.5; (**b**) *h_b_/b_b_* = 1.

Figure 7 plots the variation of the shear strength with *f_v0_*, provided by the formulations listed in Table 2 for two values of *h_b_/b_b_* (0.5 and 1) and a fixed value of *μ* = 0.58. The continuous lines refer to the equations using local parameters, i.e., Equations (7) and (8) related to the HSS failure, while the dashed lines refer to the equations using global parameters, i.e., Equations (9) and (11) related to the DSS failure. Conversely, Figure 8 plots the variation of the shear strength with *μ*, for two values of *h_b_/b_b_* (0.5 and 1) and a fixed value of *f_v0_* = 0.25 MPa.

Figure 7a,b show that for all the formulations the shear capacity increases with *f_v0_*; in the case of *h_b_/b_b_* = 0.5 (Figure 7a) the shear strengths for HSS (Equations (7) and (8)) are lower than the ones related to DSS (Equations (9)–(11)), due to a higher effect of the interlocking between units that increase the shear strength when the global parameters are used. Conversely, in the case of *h_b_/b_b_* = 1 (Figure 7b), the shear strengths based on the global parameters reduce and become lower than the shear strengths using the local ones, being the latter clearly independent of *h_b_/b_b_*. Figure 7 also shows that Equation (7) provides the highest values of the shear capacity for HSS, while Equation (10) provides the highest strengths for DSS.

Figure 8 highlights that the shear strength provided by the formulations related to the HSS failure using local parameters always increases with *μ*, while the shear strength based on global parameters (DSS failure) increases with *μ* for *h_b_/b_b_* = 0.5 (Figure 8a) and is practically constant for *h_b_/b_b_* = 1 (Figure 8b).

**Figure 9:**
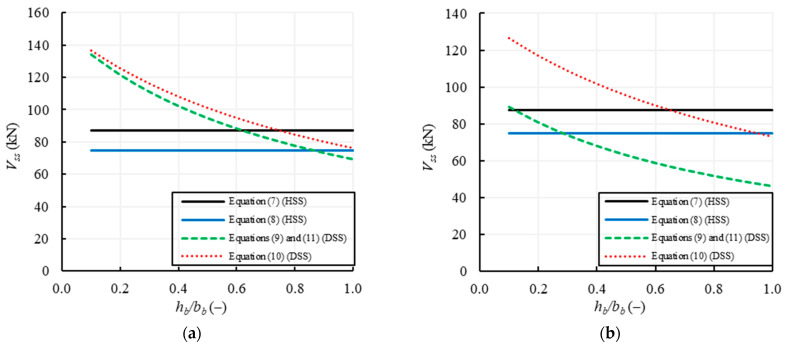
Sensitivity analysis for the shear capacity in regular masonry in function of *h_b_/b_b_* for *f_v0_* = 0.25 MPa, *μ* = 0.58, and for: (**a**) *λ* = 1; (**b**) *λ* = 1.5.

In particular, about the effect of *h_b_/b_b_* on Equations (9)–(11), Figure 9 shows that the DSS strength provided by these formulations reduces with increasing *h_b_/b_b_*, even considering two different slenderness values, i.e., *λ* = 1 in Figure 9a and *λ* = 1.5 in Figure 9b. If *h_b_/b_b_* increases, there is, indeed, a lower interlocking between the overlapped units along the horizontal mortar joints. Conversely, Equations (7) and (8) are independent of both *λ* and *h_b_/b_b_*, because the horizontal sliding failure only depends on local parameters. 

Figure 9a also shows that in the case of squat walls, i.e., *λ* = 1, Equation (8) provides safer results than Equations (9) and (11) for *h_b_*/*b_b_* < 0.85 (slender units) and, thus, a horizontal sliding shear (HSS) failure is expected, while for *h_b_*/*b_b_* > 0.85 (squat units), Equations (9) and (11) become safer than Equation (8) and, thus, a diagonal sliding shear (DSS) failure is expected. The same trend is shown in Figure 9b for slender walls, i.e., having *λ* = 1.5, but it can be noted that the threshold value of *h_b_*/*b_b_*, which indicates when the HSS failure occurs more probably that the DSS one, is reduced to 0.3. This means that for slender walls, only in the case of very slender units, i.e., with *h_b_*/*b_b_* < 0.3, Equation (8) is safer than Equations (9) and (11) and, thus, the HSS failure can occur instead of the DSS one. This occurs because Equations (9) and (11) depends on the slenderness of the wall, *λ*, through the shape factor *b*.

Anyway, these results are due to the fact that, in the formulations using the global parameters (Equations (9)–(11)), there is a significant effect of the unit interlocking and, thus, the results are very sensitive to the parameter *h_b_*/*b_b_*. It is worth noting that the relevance of the ratio *h_b_*/*b_b_*, in determining the development of horizontal (HSS) or stepped diagonal (DSS) cracks is strongly affected by the shape factor, *b*, of the wall, too. In fact, the value of the threshold, which theoretically influences the occurrence of the HSS or the DSS sliding failure mode, tends to reduce when the slenderness of the wall increases. It can be observed that in most real situations *h_b_*/*b_b_* is lower than 1 because the units are usually arranged along the longest side, and, thus, it is expected that in squat walls (λ = 1) the HSS failure is generally predominant, while in slender walls (λ = 1.5) the HSS failure can occur only for very slender units.

### 3.3. Shear Failure in Irregular/Rubble Masonry

**Figure 10:**
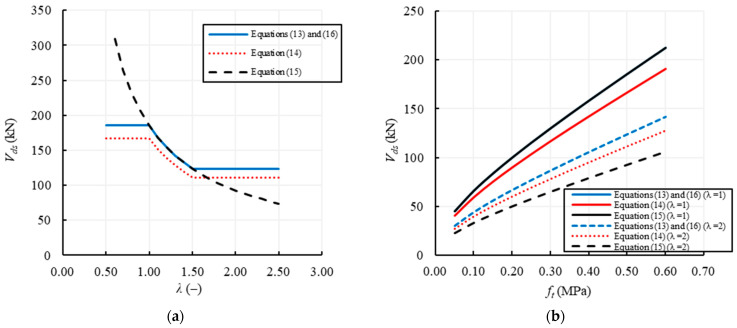
Sensitivity analysis of the shear formulations for irregular/rubble masonry in function of: (**a**) wall slenderness, *λ*, for *f_t_* = 0.5 MPa; (**b**) tensile strength of masonry, *f_t_*, in the case of *λ* = 1.0 and 2.0.

Table 3 shows that the parameters involved in the evaluation of the shear capacity for irregular/rubble masonry walls are: the geometrical parameters of the wall, *B*, *H*, *λ* and *s*, the vertical compressive stress, *σ*_0_, and the tensile strength of masonry, *f_t_*. The constraint conditions are only considered by Abrams in Equation (15) through the factor *ψ*. It is worth remembering that Equation (15) depends on *λ* and on the constraint conditions, while in Equations (13), (14) and (16) the dependence on *b* = *λ* is limited to the range 1–1.5. Again, since no difference is assumed in these comparisons between the average and the design value of the tensile strength, Equations (13) and (16) provide the same shear capacity, and, therefore, only Equation (13) is plotted in Figure 10.

Figure 10a,b show the variation of the shear capacity with the parameters *λ* and *f_t_* in the range 0.5–2.5 and 0.05–0.6 MPa, respectively. The first figure shows that Equations (13), (14) and (16) clearly provide constant values of the shear capacity for *λ* ≤ 1 and *λ* ≥ 1.5, while for Equation (15) the shear capacity is always variable with *λ*, since it directly depends on it. When the double-fixed condition is considered in Equation (15), the double aspect ratio, 2*ψλ*, is exactly equal to the shape factor, *b*, present in Equations (13) and (16), and, as a consequence, they provide the same values when *H/B* ranges in 1–1.5. As highlighted above, Equation (14) always provides 10% lower values of capacity in comparison with Equations (13) and (16), since it subtracts a 10% rate of capacity to take into account the cyclic degradation action.

Besides, Figure 10b shows that all the shear capacity curves for irregular/rubble masonry walls increase with the tensile strength, with an almost linear law. Specifically, it is highlighted how, for squat walls, i.e., *λ* = 1, Equation (15) provides the highest values and coincides with Equations (13) and (16). Again, Equation (14) provides results about 10% lower than those given by the other equations. Conversely, for slender walls, i.e., *λ* = 2, Equations (13) and (16) still provide the highest values of the shear capacity, while Equation (15) provides the lowest ones because it depends on *λ*, independently of its range of variation.

## 4. Theoretical vs. Experimental In-Plane Shear Capacity

In order to assess the reliability of the theoretical formulations described in Section 2, some experimental data for shear-compression tests on unreinforced masonry walls, subjected to constant vertical overloads and cyclic horizontal loads, have been collected and summarized in two databases, one made of regular masonry walls and the other made of irregular/rubble masonry walls. Successively, the experimental and theoretical in-plane shear capacities of masonry walls are critically compared.

### 4.1. The Experimental Database on Regular Masonry Walls

Based on the examination of the literature concerning experimental tests on regular masonry walls, a dataset of the results of shear-compression tests has been built and the main data are listed in Table 5. The first driving criterion for selecting the experimental tests has been the choice of walls constrained in a double-fixed condition in a shear-compression scheme. In all the selected experimental tests, the horizontal actions are applied to walls according to a quasi-static cyclic loading history.

The first part of the dataset collects the cases (from Case 1-R to Case 53-R) for which the complete knowledge of the geometrical and mechanical characteristics of the materials is available. Conversely, for the second part of the dataset (from Case 54-R to Case 93-R), some information, such as the dimensions of the units and/or the cohesion and the friction coefficient, is missing. This means that not all the previously discussed formulations, from Equations (7) to (12), for predicting the shear strength in regular masonry walls, can be applicable to the whole database.

For each series of the collected experimental data, the single specimens are numbered from 1 to *n*, being *n* their total number, followed by the type of masonry, ‘R’ for regular. The parameters are the same as in Table 4, with the addition of the unit shape ratio, *φ*, the volume of holes, *Ф*, when available, and the compressive strength of units, *f_bc_*. The values of all the mechanical properties are obtained by means of the experimental tests carried out by the related authors. In particular, the compressive strength of masonry, *f_c_*, was evaluated by means of experimental compressive tests on masonry wallets and *f_t_* by means of diagonal compression tests, interpreted according to the ASTM indications [23], i.e., *f_t_* = 0.707*N*/*A*, where *N* is the maximum compressive load and *A* is the area of the cross section. The values of cohesion, *f_v_*_0_, and of the friction coefficient, *µ*, when available, were evaluated experimentally according to shear tests on couplets or triplets. Moreover, the compressive strength of units, *f_bc_*, was obtained according to laboratory compressive tests on single specimens carved from the units. In the last columns of Table 5, the failure modes and the shear capacities, *V_exp_*, experimentally obtained are reported together with the corresponding experimental average shear stress defined as *τ_av_* = *V_exp_/A*.

Anthoine et al. [11] have studied the experimental in-plane behaviour of clay brick walls (Cases 1-R and 2-R in Table 5), with a base width of 1.0 m and two slenderness ratios (*λ* = 1.35 and 2.00). The main aim of this study was to analyze the effect of the slenderness on the in-plane behavior of walls. In particular, it was evidenced that under the same constraint conditions and vertical axial load, the slender wall (*λ* = 2.00) was mainly subjected to the flexural failure, while the squat wall (*λ* = 1.35) to the shear failure. Based on the results of the experimental tests of [11], Magenes and Calvi [42] have derived the formulations to predict the flexural and shear capacities, i.e., the previously described Equations (2) and (10), respectively.

Magenes et al. [64] have analyzed the experimental in-plane response of regular masonry walls made of different materials. The walls from Case 3-R to Case 6-R in Table 5 were made of calcium silicate units and those from Case 7-R to Case 10-R of hollow clay bricks. Different dimensions of the walls were considered and the slenderness, *λ*, varied between 1.0 and 2.0. The top vertical load was also varied in order to provide compressive stresses ranging from 0.5 to 2 MPa. The failure modes experimentally observed were mainly related to diagonal sliding shear (DSS) and, in some cases, to flexural failures (F). A wide variation in ductility and drift capacity was observed depending on the failure mechanism, which was influenced by all the examined parameters, i.e., the axial load, the geometry and the boundary conditions. In particular, the drift capacities were strongly affected by the type of failure and the highest values were obtained when the sliding shear failure along mortar joints occurred.

Messali et al. [65] have provided results related to regular masonry walls made of calcium silicate units (from Case 11-R to Case 14-R in Table 5), characterized by two different geometries to test both squat and slender walls. The slender walls were 1.1 m long and 2.7 m high (*λ* = 2.45), while the squat walls were 4 m long and 2.7 m high (*λ* = 0.67). The thickness of the walls was the same and equal to 0.1 m, while the top load was varied in order to have compressive stresses varying in the range 0.3–0.7 MPa. The experimental results evidenced a significant effect of the wall slenderness on the failure modes and the drift capacities of the walls. In fact, the typical failure modes of regular masonry walls were attained, i.e., HSS and DSS failures for squat and slender walls, respectively.

Morandi et al. [66] have carried out researches on the in-plane behavior of slender masonry walls made of hollow clay bricks with slenderness, *λ*, varying between 1.0 and 1.6 (from Case 15-R to Case 19-R in Table 5). The cyclical response of these walls evidenced a TDS failure, regardless of the slenderness. The results showed that, in all the cases, the cracks start developing in the units and then move towards the horizontal and vertical mortar joints.

Tomaževič [67] has carried out experimental tests on 11 unreinforced regular masonry walls made of hollow clay bricks (from Case 20-R to Case 30-R in Table 5) with slenderness varying between 1.3 and 1.5 and assuming a vertical compression stresses variable in the range 0.9–2 MPa. The walls were built using different units with a significant scattering of the mechanical properties. All the experimental tests showed a failure mechanism mainly related to the TDS failure.

Churilov et al. [68] have performed experiments with the purpose of evaluating the stiffness, the shear strength and the energy dissipation of masonry walls made of clay bricks (from Case 31-R to Case 34-R in Table 5). The tested walls had the same thickness and height, i.e., *s* = 0.25 m and *H* = 1.8 m, and two widths, i.e., *B* = 1.5 m and 2.6 m, corresponding to two slenderness values, *λ* = 0.7 and 1.2, under two different vertical loads, i.e., 0.5 and 1 MPa. The failure mechanisms observed during the experimental tests were mainly related to the TDS failure, except for the last case where a DSS failure occurred.

Salmanpour et al. [69] have carried out experimental tests on regular masonry walls made of hollow clay bricks (Case 35-R, Case 36-R and from Case 39-R to Case 43-R in Table 5) and calcium silicate units (Case 37-R and 38-R in Table 5). Different dimensions of the walls were considered and the slenderness, *λ*, varied between 0.7 and 1.5. The vertical load was also varied in order to provide compressive stresses ranging from 0.3 to 1.2 MPa. The failure modes experimentally observed were mainly related to the TDS failure and, in some cases, to the DSS failure. The experimental tests evidenced a very limited displacement capacity, regardless of the failure mode exhibited, and it was evidenced by the narrowness of the current codes to reliably assess the displacement capacity of masonry structures.

Petry and Beyer [70] have investigated the in-plane behavior of five regular masonry walls made of hollow clay bricks (from Case 44-R to Case 48-R in Table 5) with the same dimensions, i.e., *B* = 2.0 m, *H* = 2.25 m and *s* = 0.2 m. The experimental tests were conducted assuming a vertical load variable in the range 0.5–1.1 MPa. All the tests showed a TDS failure.

Morandi et al. [71] have experimentally investigated the behavior of thin walls made of hollow clay bricks. The tested masonry walls (from Case 49-R to Case 53-R in Table 5) were built with the same thickness, *s* = 0.35 m, the same height, *H* = 2.14 m, and two base widths, *B* = 1.35 m and 2.7 m, corresponding to a slenderness of *λ* = 0.8 and 1.6, respectively. The experimental tests were carried out under three levels of vertical compression stress, equal to 0.15 MPa, 0.45 MPa and 0.65 MPa. The experimentally observed failure modes were mainly related to the TDS and DSS failures.

From Case 54-R to Case 93-R in Table 5, the database is increased adding other experimental tests performed on regular masonry walls made of clay bricks [27,72], hollow clay bricks [73], lightweight aerated concrete units [64,73], calcium silicate units [73], yellow tuff stones [41,74], and dry stones [75]. The available data for these cases are not complete, as shown in Table 5.

### 4.2. Theoretical vs. Experimental Shear Capacities for Regular Masonry Walls

The comparisons between the experimental and the theoretical shear capacities of regular masonry walls are here described for the first part of the dataset reported in Table 5 (from Case 1-R to Case 53-R), because only for these tests the parameters necessary for calculating the theoretical predictions, *V_th_*, given by the formulations presented in Section 2, are available. The values of *V_th_* are listed in Table 6, together with the experimental failure loads, *V_exp_*, and modes. 

As indicated in Section 3, some formulations are plotted together because they provide the same results, assuming the average values of strength in all the calculations. In addition to the theoretical results provided by the different formulations, Table 6 also reports: (1) the minimum value of the strength provided by the formulations predicting the same failure mode observed in the tests, *V_th_*,*_min_*_,*exp*_; (2) the minimum value of the theoretical strength within all the formulations, *V_th_*,*_min_*; (3) the ratios of *V_th_*,*_min_*_,*exp*_ and *V_th_*_,*min*_ to the experimental strengths, i.e., *ρ_min_*_,*exp*_ and *ρ_min_*, respectively. Note that for *ρ_min_* the corresponding theoretical failure mode is reported in brackets too.

It is important to note that among all the formulations for the shear strength for regular masonry walls discussed in Section 2.2.1, only Equation (12), which provides the diagonal shear strength for the tensile failure (TDS), uses the tensile strength of the units, *f_bt_*. Since this parameter was not provided by the authors and in order to calculate Equation (12), *f_bt_* has been evaluated by means of the correlation proposed by Eurocode 6 [55]: *f_bt_* = 0.032*f_bc_*, being *f_bc_* the compressive strength of the masonry unit. Such a formulation seems to be more realistic in comparison with the one suggested by the Commentary to the Italian code [54], which provides *f_bt_* = 0.1*f_bc_* and tends to overestimate too much the experimental TDS capacities of the wall. Conversely, the equation proposed by Eurocode 6 [55] leads to theoretical TDS capacities more comparable with the experimental ones.

Table 6 shows that the failure modes associated with the minimum capacities, *V_th_*_,*min*_, agree with the experimentally observed ones in most of the cases. The main differences with the experimental results are provided by Equations (9)–(12), especially when the related theoretical values are comparable. In some cases, in fact, the predicted failure mode is DSS, while the experimentally observed one is TDS or vice versa. However, this disagreement can be explained because the experimentally observed failure modes were often characterized by cracks initially starting from the units and, then, moving across the mortar joints, or vice versa. This is confirmed by the comparable values of the DSS and TDS strengths and means that the activation of one or the other failure mode as the first one is also related to the experimental scatter of the mechanical properties of the material within the wall.

The experimental failure modes also confirm that the slenderness of the walls, *λ*, and of the units, *h_b_/b_b_*, influence the prevalence of the DSS or the HSS failure mode (Figure 9), as already discussed in Section 3.2.

**Figure 11:**
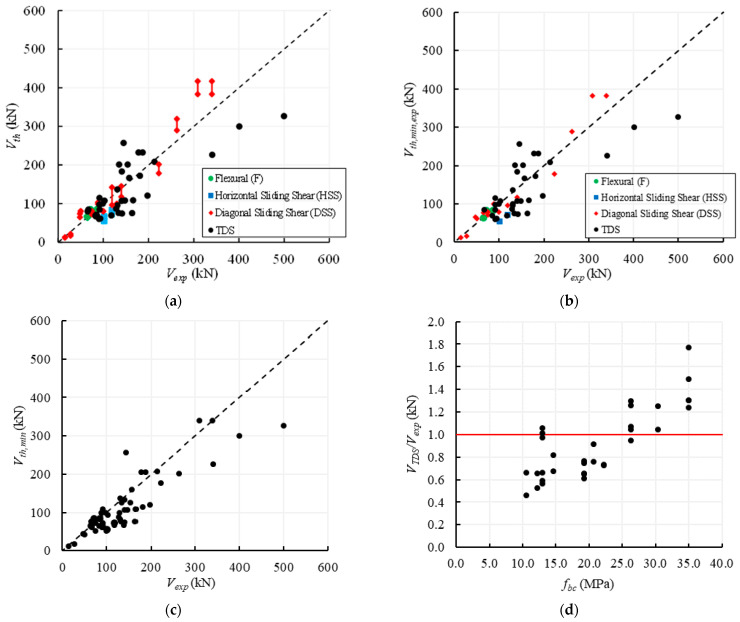
Experimental vs. theoretical results calculated as: (**a**) ranges of strengths related to the same failure mode observed in the experimental tests; (**b**) minimum strength corresponding to the experimental failure mode, *V_th_*_,*min*,*exp*_; (**c**) minimum strength within the theoretical results, *V_th_*_,*min*_; (**d**) theoretical-to-experimental TDS capacity vs. compressive strength of units.

Figure 11 shows the comparisons between the experimental and the theoretical values of the shear capacity. In particular, in Figure 11a, the experimental failure loads are compared with the ranges of strengths provided by all the formulations predicting the same failure modes observed in the tests. In Figure 11b, each experimental value is only compared with *V_th_*,*_min_*_,*exp*_, which is the minimum strength provided by all the formulations predicting the same failure mode experimentally observed. In Figure 11c the minimum values of strengths, *V_th_*,*_min_*, within all the theoretical results provided by the Equations (1)–(12) listed in Table 6 are considered, independently of the experimental failure mode. Finally, Figure 11d shows the correlation between the theoretical-to-experimental TDS capacities and the compressive strength of the unit, *f_bc_*.

In order to numerically define the differences between the experimental and the theoretical capacities, the mean, the standard deviation and the coefficient of variation (CoV) relative to the factors *ρ_min_*_,*exp*_ and *ρ_min_* are calculated and listed in Table 7. It is worth highlighting that these factors are referred to the theoretical values plotted in Figure 11b,c, respectively.

More in detail, Figure 11a shows that in the cases of the flexural failure, the agreement between the experimental results and theoretical predictions is very good and there is a very low scattering among the values provided by the different theoretical formulations, as already observed in Section 3.1. In fact, for the 3 specimens that attained a flexural failure the average value of the theoretical capacities is equal to the experimental one.

Conversely, in the cases of the HSS failure, Equations (7) and (8) significantly underestimate the experimental results (57% on average), but only 2 specimens attained such a failure mode and, thus, the results are not statistically significant. The underestimation of the horizontal sliding shear capacities may also be related to the assumption of a reduced length of the cross section equal to half length of the specimen, i.e., *B*’ = 0.5*B*.

Finally, Figure 11a shows that for the 15 specimens failed for DSS, there is a significant variability among the results provided by Equations (9)–(11), as already observed in Section 3.2. For these specimens, it is highlighted that Equations (9) and (11) provide an average value of the theoretical capacities very close to the ones experimentally obtained, while Equation (10) tends to overestimate the actual shear capacity of about 15%.

However, if only the minimum values of strengths associated with the same experimentally observed failure modes are plotted, Figure 11b shows that the agreement between these theoretical and experimental results is quite good. This agreement is already evidenced by the average value of *ρ_min_*_,*exp*_ (0.94, CoV = 30.4%) and is mainly due to the higher reliability of Equations (9), (11) and (12) into predicting the DSS and TDS failures, respectively. Besides, no substantial differences can be observed for the flexural failure, while for HSS the few experimental results are underestimated by both Equations (7) and (8). Figure 11b also shows that in most cases the minimum values of the theoretical formulations associated with the same experimental failure are safe or close to the experimental strength.

If the actual failure mode is not known ‘a priori’, the prediction of the strength is based on the assessment of the minimum value within the strengths associated with different failure modes; therefore, in Figure 11c, the minimum values of strength among all the theoretical predictions are compared with the experimental results. It can be noted that the graph is similar to Figure 11b because in few cases the ‘predicted’ failure mode does not correspond to the experimental one, while the theoretical predictions are conservative, i.e., the average value of *ρ_min_* is 0.82 (CoV = 29.4%).

Finally, focussing on the 33 specimens failed for TDS, it can be observed that Equation (12) provides theoretical capacities that are on average only 8% lower than the experimental ones, i.e., the average value of the ratio *V_th_*_,*TDS*_/*V_exp_* is 0.92, but the CoV is 34.3%. Figure 11d shows that the theoretical predictions for TDS are safe until the compressive strength of the units is lower than 25 MPa; for higher values of *f_bc_*, also the correlation suggested by Eurocode 6 [55], i.e., *f_bt_* = 0.032*f_bc_*, becomes not safe, since Equation (12) tends to overestimate the experimental capacities too much.

In conclusion, it can be derived that the flexural capacities for regular masonry walls can be reliably provided by the available formulations, while the shear strength for DSS is safely predicted by Equation (9) proposed by Mann and Muller [58], which is the same as Equation (11) recalled in the Italian code [53]. For the HSS failure, Equation (7) seems to be quite reliable in predicting the experimental results because it provides a low underestimation, but the available data are not enough to obtain conclusive remarks. For the TDS failure, Equation (12) provides safe values of the shear strengths if the tensile strength of the unit is calculated as about 3% of the compression strength of the unit, according to Eurocode 6 [55], and, however, for compressive strength not higher than 25 MPa.

When the failure mode is not known ‘a priori’, the wall strength can reasonably be predicted by the minimum value among the ones provided by Equations (3), (8), (9), (11) and (12).

### 4.3. The Experimental Database on Irregular/Rubble Masonry Walls

For irregular/rubble masonry walls, the available formulations for the shear capacity can all be referred to the Turnšek and Čačovič approach [14], which is only based on the geometrical parameters of the wall and the tensile strength of masonry, assumed as a homogeneous material. Thus, the formulations for irregular/rubble masonry walls are simpler to be applied than the ones based on the Mohr-Coulomb criterion for regular masonry, and some codes, i.e., the Commentary to the Italian code [54], allow to use them in favor of safety for predicting the diagonal shear strength also in walls made of regular masonry. This aspect will be addressed in detail in Section 4.5.

Regarding the irregular/rubble masonry walls, the experimental tests carried out by [10,26,27,76,77,78] are herein considered to define a database of a total of 27 tests. In particular, the selected experiments were performed according to shear-compression tests on walls made of single-leaf stones [10,26,27,76,78], double-leaf stones [27,76,78] and three-leaf stones [77]. The constraint condition of the walls was in all the cases ‘double-fixed’ and quasi-static cyclic actions were applied in all the testing procedures. The slenderness, *λ*, varies from 1.0 to 2.0 and the normal compressive stress, *σ_0_*, in the range 0.1–1.5 MPa. The compressive strength, *f_c_*, of the masonry varies in 2.5–21.0 MPa, while the tensile strength, *f_t_*, obtained according to the ASTM indication, i.e., *f_t_* = 0.707N/A, varies in 0.04–0.25 MPa. The compressive strength of the unit, *f_bc_*, was evaluated by means of experimental compressive tests and varies from 7.5 MPa to 165.0 MPa. In Table 8 the main geometrical and mechanical parameters of the walls are summarized, together with the experimental failure modes, loads and average shear stress. It can be observed that, as expected for such a masonry typology, all the collected walls failed for diagonal shear with cracks in the units and/or in the mortar (i.e., DS failure, Figure 1b).

### 4.4. Theoretical vs. Experimental Capacities for Irregular/Rubble Masonry Walls

As indicated in Section 2, irregular/rubble masonry walls can exhibit only one type of shear failure, i.e., diagonal shear (DS). The theoretical shear strengths are herein evaluated for the tests collected in Table 8 by means of the formulations usually adopted for diagonal shear failure in irregular/rubble masonry walls, i.e., Equations (13)–(16). Thus, in Table 9, the experimental maximum loads, *V_exp_*, and the theoretical capacities, *V_th_*_,*DS*_, are listed, together with their ratio, *ρ_DS_*. The theoretical flexural capacities provided by Equation (3), i.e., the minimum ones within all the formulations predicting the flexural failure, *V_th_*_,*F*_, are also listed with their ratio, *ρ_F_* = *V_th_*_,*F*_*/V_exp_*. It can be observed that the theoretical flexural strengths are in most cases greater than the experimental failure loads (average value of *ρ_F_* is 1.64 with CoV = 30.4%) and always greater than the diagonal shear strengths provided by Equations (13) and (16), confirming, thus, the experimental occurrence of the DS failure.

**Figure 12:**
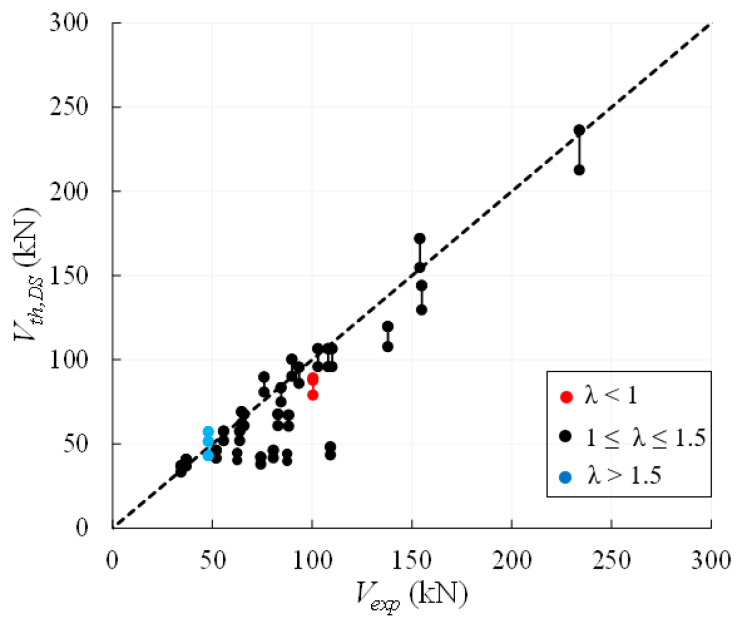
Experimental vs. theoretical results for irregular masonry walls collected by slenderness.

The comparison between the experimental and theoretical values of the shear strength of irregular/rubble masonry walls is plotted in Figure 12, collecting the 27 examined walls into three groups of slenderness values, i.e., *λ* < 1, 1 ≤ *λ* ≤ 1.5, λ > 1.5. Such a distinction is related to the fact that Equations (13)–(16) provide the same strength values when 1 ≤ *λ* ≤ 1.5 and different values for *λ* < 1 or *λ* > 1.5 (see Figure 10a). Figure 12 shows that the formulations are mostly conservative and, in particular, Equation (14) is always the safest one due to the 10% reduction of the strength related to cyclic actions. For Equation (14), the average value of *ρ_DS_ = V_th_*_,*DS*_*/V_exp_* is, indeed, 0.83 with a CoV = 22.4%. Provisions given by Equations (13), (15) and (16) are practically coincident since most walls (25 out of 27) have slenderness 1 ≤ *λ* ≤ 1.5; the average value of *ρ_DS_* is, thus, about 0.91, i.e., about 10% higher than the value associated to Equation (14), with a similar CoV.

### 4.5. Reliability of Diagonal Shear Design Formulations for Regular Masonry Walls

In order to check the safety of using the design formulations for the diagonal shear failure of irregular/rubble masonries also for regular masonry walls, as suggested by the Commentary to the Italian code [54], a further comparison with the collected experimental results is presented in Table 10.

In this table, the whole database for regular masonry walls reported in Table 5 is considered, with the exception of the three specimens that attained a flexural failure. Independently of the experimental failure modes, the maximum experimental loads are compared with the theoretical shear capacities, *V_th_*_,*DS*_, provided by Equations (13)–(16), usually adopted for predicting the DS failure of irregular/rubble masonry walls. The single values of the theoretical-to-experimental shear strength ratios, *ρ_DS_*, with reference to the DS strength values provided by Equations (13)–(16) are also reported.

Finally, in Table 10, the minimum value within all the theoretical-to-experimental shear strength ratios, *ρ_min_*, calculated with all the formulations, i.e., from Equations (1) to (16) valid for regular or irregular masonry walls, is listed too for the only cases from 1-R to 53-R for which all the data are available. For the other tests, i.e., from 54-R to 93-R, such a ratio is not listed since not all the theoretical formulations can be calculated. For each value of *ρ_min_*, the failure mode corresponding to the minimum theoretical strength is reported in brackets. It can be noted that, because the theoretical failure modes are always due to DSS, HSS or TDS, the strength models for regular masonry walls, i.e., from Equations (13) to (16), are always safer than those provided for irregular masonry ones.

In Table 11, the average value, the standard deviation and the coefficient of variation (CoV) of the ratio *ρ_DS_* are listed for the three groups of equations and for the three ranges of *λ*.

The values of *ρ_DS_* in Table 10 evidence that the theoretical diagonal shear capacities are greater than the experimental ones since they vary in average in the range 1.10–1.23. Moreover, as previously discussed, they are generally greater than *ρ_min_* provided by the formulations for regular masonry.

The minimum values of *ρ_DS_* are provided by Equation (15) for slender walls, i.e., for *λ* > 1.5, and by Equation (14) for squat walls, i.e., for *λ* ≤ 1.5, in agreement with the sensitivity analyses discussed in Section 3. This result can be explained by considering that the shear capacities provided by Equations (13), (14) and (16)) are affected by the shape factor, *b*, which is limited in the range 1.0–1.5, while Equation (15) is influenced by the aspect ratio, *ψλ*, without any limitation. Note that Equation (14) considers a 10% reduction with respect to Equations (13) and (16) to take into account cyclic loads and, thus, it should be more reliable than the other formulations because cyclic loads are applied in all the tests of the collected database.

**Figure 13:**
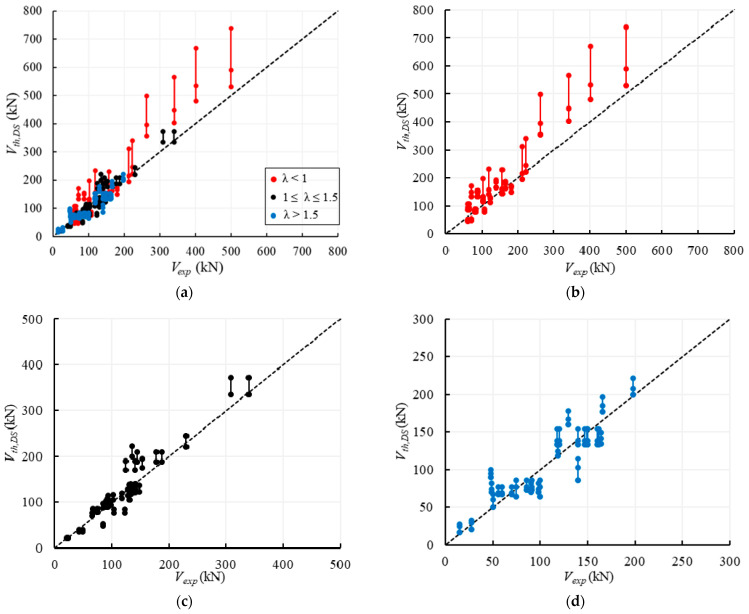
Experimental vs. theoretical results for regular masonry walls: (**a**) all data; (**b**) *λ* < 1; (**c**) 1 ≤ *λ* ≤ 1.5; (**d**) *λ* > 1.5.

The comparisons between the experimental and theoretical strengths provided by Equations (13)–(16) are firstly plotted in Figure 13a with reference to the whole database of regular masonry walls, collected by different ranges of slenderness; successively, the data are plotted with reference to each range of slenderness in order to better compare the results. For walls with *λ* ≤ 1.5 (Figure 13a,b), the theoretical formulations tend to overestimate the experimental shear capacities, while a better agreement is observed for slender walls, i.e., for *λ* > 1.5 (Figure 13c).

Table 10 and Table 11 and Figure 13 highlight that Equations (13) and (16), i.e., the same formulation proposed by Turnšek and Čačovič [14] and the Italian code [53], provide the highest values of the shear capacities for *λ* ≥ 1, since their average value of *ρ_DS_* is equal to 1.09 (CoV =18.3%) for 1 ≤ *λ* ≤ 1.5 and to 1.17 (CoV = 25.1%) for *λ* > 1.5. Conversely, for *λ* < 1, Equation (15) provides the highest predictions with an average value of *ρ_DS_* equal to 1.45 (CoV = 28.1%). Finally, Table 10 and Table 11 also highlight that Equation (14), provided by Tomaževič and Lutman [50], is the safest one independently of the wall slenderness, since it provides the lowest average value of *ρ_DS_*, which is 1.03 (CoV = 22.7%) for all tests.

#### A Proposal for Correcting the Shape Factor b

**Figure 14:**
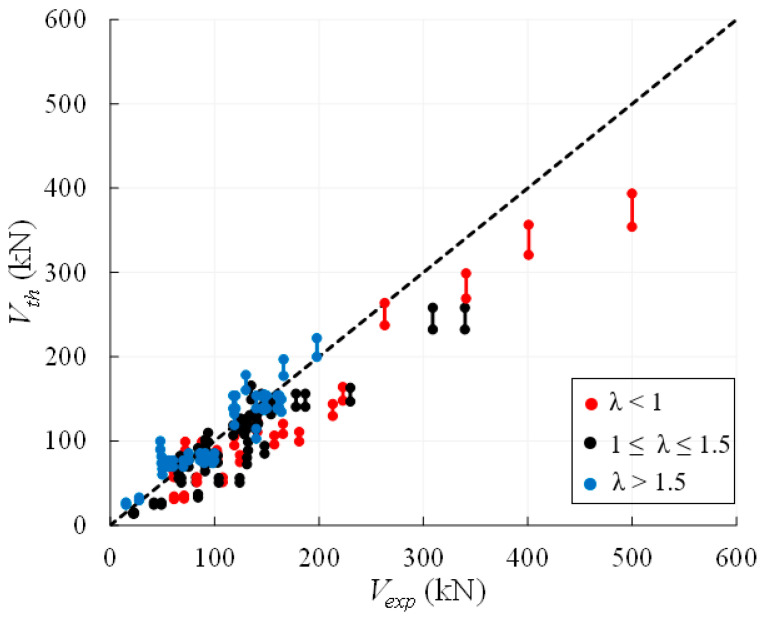
Experimental vs. theoretical results of regular masonry walls provided by Equations (13), (14) and (16) assuming *b* = 1.5.

Considering that Equations (13), (14) and (16) are affected by the shape factor *b*, if *b* is assumed equal to 1.5 also for squat walls, which are the majority part of the collected database, the theoretical values reduce and, thus, become safer on average. This is highlighted in Figure 14, where the same experimental loads plotted in Figure 13 are compared with the results provided by Equations (13) and (16) and Equation (14) modified with the assumption *b* = 1.5 for whatever value of the wall slenderness. Note that when *b* = 1.5 is assumed in Equations (14) and (16), these Equations will be labelled in the following as as (14)* and (16)*.

The mean, the standard deviation and the CoV associated with the already defined ratio *ρ_DS_*, but calculated with reference to Equations (13) and (16)* and Equation (14)*, being the latter ones modified assuming *b* = 1.5, are summarized in Table 12.

It can be noted that Equation (14)* always provides the lowest values of the shear capacities with an average value of *ρ_DS_* = 0.85 for all tests (CoV = 29.3%), while Equations (13) and (16)* provides an average value of *ρ_DS_* equal to 0.94. These values correspond to a reduction by about 18% with respect to the ones listed in Table 11 and show that the assumption on the shape factor allows the results are safer and more reliable for both the theoretical predictions. It is worth noting that, with respect to the values listed in Table 11, the correction proposed for the factor *b* only influences the values of *ρ_DS_* concerning the cases with *λ* ≤ 1.5.

Finally, it is worth noting that the average value of *ρ_DS_* and the CoV related to Equation (14)*, i.e., 0.85 and 29.3%, are comparable with the values associated with the minimum shear strength for regular masonry walls, i.e., 0.82 and 29.4% (see Table 7 and Table 11), though they are calculated on a smaller database (93 vs. 53 data). This should imply that in the lack of information about the cohesion and the friction angles for regular masonry walls, a reliable prediction of their shear strength can be represented by the formulations assessing the diagonal shear failure of irregular/rubble masonry walls, based on the Turnšek and Čačovič model [14] if the shape factor *b* is assumed equal to 1.5 for whatever value of wall slenderness.

## 5. Conclusions

In this research work, an in-depth literature review of the theoretical formulations used to predict the in-plane capacities of masonry walls is reported with reference to both flexural and shear failures. For shear failures, the literature formulations were classified according to the types of masonry, i.e., regular and irregular/rubble, because different failure mechanisms may activate depending on the masonry types. Walls made of regular masonry are, indeed, mainly subjected to a sliding shear failure according to horizontal cracks along adjacent bed joints (HSS) or to diagonal stepped cracks involving both vertical and horizontal mortar joints (DSS); in both cases, the parameters influencing the shear strength are related to sliding phenomena and, thus, to the assessment of the cohesion and the friction angle. On the other hand, irregular/rubble masonry walls mainly exhibit a diagonal shear failure with cracks crossing both the units and the mortar joints (DS) and the shear strength is related to the tensile strength of masonry. Moreover, a diagonal shear for the tensile failure of the units (TDS) can also be observed for regular masonry walls.

Sensitivity analyses were carried out to investigate the effect of the main parameters on the different literature strength models and to quantify the variability among the formulations. In particular, slight differences were observed among the formulations used to predict the flexural capacities of both regular and irregular masonry walls. Conversely, the shear capacities of regular walls are strongly dependent on the parameters used to identify the shear properties of masonry, i.e., local or global values of the cohesion and the friction coefficient. The values of the shear strength associated with horizontal or diagonal sliding phenomena, i.e., HSS and DSS failure modes, are different and, also within the same strength model, different formulations are available in the literature. Moreover, it was observed that the dimensions of the units have a significant effect on the failure modes and loads because it influences the global properties of masonry and, coupled with the wall slenderness, may drive the sliding shear failure mode towards the HSS or the DSS failure. Concerning the irregular/rubble masonry walls, the available formulations for predicting the diagonal shear failure have similar trends, because all depend on the same parameters, with the exception of the wall slenderness, which is limited to the range 1.0–1.5 in one formulation and can attain whatever value in the other ones.

An accurate literature review of several experimental shear-compression tests on masonry walls was carried out to collect a database of experimental results, with reference to both regular and irregular/rubble masonry walls. The collected experimental results (120 data in total) were compared with the outcomes from the literature formulations for flexural and shear strength models to verify their reliability.

Firstly, a reduced sample of regular masonry walls (53 data), for which all the data necessary for calculating the flexural and sliding shear strengths were available, was considered. The comparisons between experimental and theoretical results evidenced a good agreement in the case of flexural failure, while, in the case of shear failure, the DSS strength is safely predicted by the strength model proposed by Mann and Muller and recalled by the current Italian code too. For the HSS failure, the classical Mohr-Coulomb criterion underestimates the experimental results, but the available experimental data are not enough to obtain conclusive remarks. Concerning the unique formulation proposed to predict the TDS failure, the main issue is the evaluation of the tensile strength of the units, which is often a missing parameter. If the correlation between the tensile and compressive strengths provided by the Eurocode 6 is assumed, i.e., the tensile strength of the units equal to about 3.2% of the compressive one, reliable predictions of the TDS failure loads for the walls that experimentally attained such a failure mode were obtained. Finally, when the failure mode is not known ‘a priori’, it was evidenced that the in-plane strength of regular masonry walls and the corresponding failure mode can reasonably be predicted by the minimum among all the values provided by the formulations for the DSS, HSS and TDS failures.

Successively, for the whole database made of both regular and irregular/rubble masonry, the experimental results were compared with the formulations predicting the DS failure usually adopted for irregular/rubble masonry, i.e., the formulations based on the Turnšek and Čačovič model. Such an expression uses the only tensile strength of masonry as a mechanical parameter and, being simpler to be used, is suggested by some codes for predicting the shear strength in walls made of regular masonry too. The comparisons evidenced that for irregular/rubble masonry walls the theoretical predictions are safe in most cases, while the contrary occurs for regular masonry walls. However, since their reliability is influenced by the value of the shape factor *b*, it was verified that, under the assumption of *b* = 1.5, these formulations become reasonably safer for regular masonry too. Moreover, it was found that both the average values of the theoretical-to-experimental load ratio and the associated CoV referred to the modified Turnšek and Čačovič formulation are comparable with the values associated with the minimum shear strength given by the specific formulations for regular masonry walls. This should imply that in the lack of information about the cohesion and the friction angles for regular masonry walls, a reliable prediction of their shear strength can be represented by the Turnšek and Čačovič formulation if *b* is assumed 1.5 for whatever value of the wall slenderness.

The outcomes of this critical literature review will be very useful to any further investigation on the in-plane behavior of masonry walls and future work will be carried out, using finite element analysis, to better investigate the effect of the mechanical properties herein discussed.

## Figures and Tables

**Table 1:** Literature formulations used to predict the flexural capacity.

Authors	Formula		Compressive Strength	Aspect Ratio
Tomaževič and Lutman [50]	Equation (1)	$V_{T,f}=Bs\frac{\sigma_{0}}{2\psi \lambda }\cdot \left(1-\frac{\sigma_{0}}{f_{c,d}}\right)$	*f_c_*_,*d*_	$\psi \lambda =\psi \frac{H}{B}$ ψ = 1 for cantilever; ψ = 0.5 for double-fixed constraint.
Magenes and Calvi [42]	Equation (2)	$V_{MC,f}=Bs\frac{\sigma_{0}}{2\psi \lambda }\cdot \left(1-\frac{\sigma_{0}}{0.85f_{c,av}}\right)$	0.85 *f_c_*_,*av*_	
Abrams [51]	Equation (3)	$V_{A,f}=Bs\frac{\sigma_{0}}{2\psi \lambda }\cdot \left(1-\frac{\sigma_{0}}{0.7f_{c,av}}\right)$	0.70 *f_c_*_,*av*_	$\psi \lambda =\frac{H_{eff}}{B}$ For H_eff_ see Figure 2 H_eff_ = H for cantilever; H_eff_ = 0.5 H for double-fixed constraint.
Eurocode 8-Part 3 [52]	Equation (4)	$V_{EC,f}=Bs\frac{\sigma_{0}}{2\psi \lambda }\cdot \left(1-\frac{\sigma_{0}}{0.87\;f_{c,d}}\right)$	0.87 *f_c_*_,*d*_	
NTC 2018 [53]	Equation (5)	$V_{NTC,f}=Bs\frac{\sigma_{0}}{2\psi \lambda }\cdot \left(1-\frac{\sigma_{0}}{0.85f_{c,d}}\right)$	0.85 *f_c_*_,*d*_	

**Table 2:** Literature and code formulations used to predict the shear capacity of regular masonry walls.

Parameters	Authors	Formula		Failure
Local for masonry *f_v0_* and *µ*	Grimm [57]	Equation (7)	$V_{G,hss}=B^{\prime }s\cdot \left(1.4f_{v0}+\mu \sigma_{0}\right)$	Sliding shear along horizontal cracks (HSS)
	Eurocode 6 [55] NTC 2018 [53]	Equation (8)	$V_{EC,hss}=B^{\prime }s\cdot \frac{f_{v0}+\mu \sigma_{0}}{\gamma_{d}}$	
Global for masonry $f_{v0}^{\prime }=\frac{f_{v0}}{1+\mu \varphi }$ $\mu^{\prime }=\frac{\mu }{1+\mu \varphi }$	Mann and Muller [58]	Equation (9)	$V_{MM,dss}=\frac{Bs}{b}\cdot \left(f_{v0}^{\prime }+\mu^{\prime }\sigma_{0}\right)$ $1\leq b=\frac{H}{B}\leq 1.5$	Sliding shear along diagonal stepped cracks (DSS)
	Magenes and Calvi [42]	Equation (10)	$V_{MC,dss}=Bs\cdot \left(\frac{1.5f_{v0}^{\prime }+\mu^{\prime }\sigma_{0}}{1+3\frac{f_{v0}^{\prime }\psi \lambda }{\sigma_{0}}}\right)$ ψ = 1 for cantilever; ψ = 0.5 for double-fixed constraint.	
	Commentary to the Italian code [54]	Equation (11)	$V_{C,dss}=\frac{Bs}{b}\cdot \left(f_{v0}^{\prime }+\mu^{\prime }\sigma_{0}\right)\leq V_{t,lim}$ $1\leq b=\frac{H}{B}\leq 1.5$	
Single units	Commentary to the Italian code [54]	Equation (12)	$V_{t,lim}=\frac{Bsf_{bt,d}}{2.3b}\cdot \sqrt{1+\left(\frac{\sigma_{0}}{f_{bt,d}}\right)}$	Diagonal shear for tensile cracking of units (TDS)

**Table 3:** Literature and code formulations used to predict the DS capacity of irregular/rubble masonry walls.

Authors	Formula		Tensile Strength	Shape Factor
Turnšek and Čačovič [14]	Equation (13)	$V_{NTC,ds}=Bs\frac{f_{t,av}}{b}\cdot \sqrt{1+\frac{\sigma_{0}}{f_{t,av}}}$	$f_{t,av}$	$1\leq b=\frac{H}{B}\leq 1.5$
Tomaževič and Lutman [50]	Equation (14)	$V_{T,ds}=0.9Bs\frac{f_{t,d}}{b}\cdot \sqrt{1+\frac{\sigma_{0}}{f_{t,d}}}$	$f_{t,d}$	
Abrams [51]	Equation (15)	$V_{A,ds}=Bs\frac{f_{t,d}}{2\psi \lambda }\cdot \sqrt{1+\frac{\sigma_{0}}{f_{t,d}}}$	$f_{t,d}$	$\psi \lambda =\frac{H_{eff}}{B}$ For H_eff_ see Figure 2 H_eff_ = H for cantilever; H_eff_ = 0.5 H for double-fixed constrain.
Commentary to the Italian code [54]	Equation (16)	$V_{C,ds}=Bs\frac{f_{t,d}}{b}\cdot \sqrt{1+\frac{\sigma_{0}}{f_{t,d}}}$	$f_{t,d}$	$1\leq b=\frac{H}{B}\leq 1.5$

**Table 4:** Mechanical and geometrical properties of masonry walls for the reference case.

*B*	*H*	*λ*	*s*	*b_b_*	*h_b_*	*σ*_0_	*f_t_*	*f_c_*	*f_v0_*	*μ*
(m)	(m)	(−)	(m)	(m)	(m)	(MPa)	(MPa)	(MPa)	(MPa)	(−)
1.00	1.00	1.0	0.25	0.15	0.15	0.60	0.50	5.00	0.25	0.58

**Table 5:** Database of experimental tests on regular masonry walls.

Case	Authors	Type of Masonry	*B*	*H*	*λ*	*s*	*b_b_*	*h_b_*	*h_b_/b_b_*	*φ*	*Ф*	*σ*_0_	*f_t_*	*f_c_*	*f_v0_*	*μ*	*f_bc_*	Failure Mode	*V_exp_*	*τ_av_*
			(mm)	(mm)	(−)	(mm)	(mm)	(mm)	(−)	(−)	(%)	(MPa)	(MPa)	(MPa)	(MPa)	(−)	(MPa)		(kN)	(MPa)
1-R	Anthoine et al., 1994 [11]	Clay brick	1000	1350	1.35	250	300	125	0.42	0.83	0	0.60	0.25	6.20	0.23	0.58	24.40	DSS	75.0	0.30
2-R			1000	2000	2.00	250	300	125	0.42	0.83	0	0.60	0.25	6.20	0.23	0.58	24.40	F	65.0	0.26
3-R	Magenes et al., 2008 [64]	Calcium silicate	1250	2500	2.00	175	248	248	1.00	2.00	0	1.00	0.27	24.00	0.60	0.54	15.10	DSS	75.0	0.34
4-R			1250	2500	2.00	175	248	248	1.00	2.00	0	0.50	0.27	24.00	0.60	0.54	15.10	DSS	50.0	0.23
5-R			1250	2500	2.00	175	248	248	1.00	2.00	0	2.00	0.27	24.00	0.60	0.54	15.10	DSS	140.0	0.64
6-R			1250	2500	2.00	175	248	248	1.00	2.00	0	1.00	0.27	24.00	0.60	0.54	15.10	DSS	100.0	0.46
7-R	Magenes et al., 2008 [64]	Hollow clay brick	2500	2600	1.04	300	250	190	0.76	1.52	45	0.68	0.28	9.50	0.60	0.54	26.50	DSS	309.0	0.41
8-R			2500	2600	1.04	300	250	190	0.76	1.52	45	0.68	0.28	9.50	0.60	0.54	26.50	DSS	340.0	0.45
9-R			1250	2600	2.08	300	250	190	0.76	1.52	45	0.50	0.28	9.50	0.60	0.54	26.50	F	86.0	0.23
10-R			1250	2600	2.08	300	250	190	0.76	1.52	45	0.50	0.28	9.50	0.60	0.54	26.50	F	72.0	0.19
11-R	Messali et al., 2020 [65]	Calcium silicate	1100	2700	2.45	102	434	476	1.10	2.19	0	0.70	0.21	5.93	0.14	0.43	27.40	DSS	27.7	0.25
12-R			1100	2700	2.45	102	434	476	1.10	2.19	0	0.40	0.21	5.93	0.14	0.43	27.40	DSS	15.0	0.13
13-R			4000	2700	0.68	102	434	476	1.10	2.19	0	0.50	0.21	5.93	0.14	0.43	27.40	HSS	119.0	0.29
14-R			4000	2700	0.68	102	434	476	1.10	2.19	0	0.30	0.21	5.93	0.14	0.43	27.40	HSS	102.0	0.25
15-R	Morandi et al., 2013 [66]	Hollow clay brick	1250	2000	1.60	350	250	190	0.76	1.52	45	0.50	0.41	9.50	0.69	0.77	19.20	TDS	130.0	0.30
16-R			1250	2000	1.60	350	250	190	0.76	1.52	45	0.70	0.41	9.50	0.69	0.77	19.20	TDS	166.0	0.38
17-R			1250	2000	1.60	350	250	190	0.76	1.52	45	1.00	0.41	9.50	0.69	0.77	19.20	TDS	198.0	0.45
18-R			2500	2000	0.80	350	250	190	0.76	1.52	45	0.50	0.41	9.50	0.69	0.77	19.20	TDS	401.0	0.46
19-R			2500	2000	0.80	350	250	190	0.76	1.52	45	0.70	0.41	9.50	0.69	0.77	19.20	TDS	500.0	0.57
20-R	Tomaževič, 2009 [67]	Hollow clay brick	1000	1430	1.43	280	188	189	1.01	2.01	58	1.92	0.23	4.88	0.27	0.40	20.70	TDS	140.6	0.50
21-R			1000	1430	1.43	280	188	189	1.01	2.01	58	0.96	0.23	4.88	0.27	0.40	20.70	TDS	92.0	0.33
22-R			1020	1510	1.48	280	238	234	0.98	1.97	55	1.71	0.24	4.89	0.26	0.40	13.00	TDS	133.7	0.47
23-R			1020	1510	1.48	280	238	234	0.98	1.97	55	0.94	0.24	4.89	0.26	0.40	13.00	TDS	90.9	0.32
24-R			1020	1510	1.48	280	238	234	0.98	1.97	55	1.37	0.24	4.89	0.26	0.40	13.00	TDS	118.0	0.41
25-R			1010	1420	1.41	290	189	188	0.99	1.99	53	1.67	0.20	4.51	0.20	0.40	14.60	TDS	128.7	0.44
26-R			1010	1420	1.41	290	189	188	0.99	1.99	53	0.89	0.20	4.51	0.20	0.40	14.60	TDS	84.2	0.29
27-R			990	1420	1.43	290	331	189	0.57	1.14	54	1.62	0.26	4.76	0.38	0.40	12.20	TDS	141.7	0.49
28-R			990	1420	1.43	290	331	189	0.57	1.14	54	1.00	0.26	4.76	0.38	0.40	12.20	TDS	93.9	0.33
29-R			1070	1470	1.37	250	254	121	0.48	0.95	25	1.96	0.23	5.44	0.33	0.40	30.30	TDS	131.0	0.49
30-R			1070	1470	1.37	250	254	121	0.48	0.95	25	1.10	0.23	5.44	0.33	0.40	30.30	TDS	91.6	0.34
31-R	Churilov et al., 2013 [68]	Clay brick	2600	1800	0.69	250	250	65	0.26	0.52	0	1.00	0.10	3.60	0.00	0.66	6.80	TDS	213.2	0.33
32-R			1500	1800	1.20	250	250	65	0.26	0.52	0	1.00	0.10	3.60	0.00	0.66	6.80	TDS	99.1	0.26
33-R			2600	1800	0.69	250	250	65	0.26	0.52	0	0.50	0.10	3.60	0.00	0.66	6.80	TDS	157.4	0.24
34-R			1500	1800	1.20	250	250	65	0.26	0.52	0	0.50	0.10	3.60	0.00	0.66	6.80	DSS	65.5	0.17
35-R	Salmanpour et al., 2015 [69]	Hollow clay brick	1500	1600	1.07	150	290	190	0.66	1.31	42	0.64	0.25	6.40	0.26	0.48	26.30	TDS	91.0	0.40
36-R			1500	1600	1.07	150	290	190	0.66	1.31	42	0.96	0.25	6.40	0.26	0.48	26.30	TDS	103.0	0.46
37-R	Salmanpour et al., 2015 [69]	Calcium silicate	1550	1600	1.03	150	250	190	0.76	1.52	25	0.77	0.26	7.70	0.26	0.48	22.20	TDS	131.0	0.56
38-R			1550	1600	1.03	150	250	190	0.76	1.52	25	1.16	0.26	7.70	0.26	0.48	22.20	TDS	148.0	0.64
39-R	Salmanpour et al., 2015 [69]	Hollow clay brick	2700	2600	0.96	150	290	190	0.66	1.31	42	0.58	0.25	6.40	0.26	0.48	26.30	TDS	141.0	0.35
40-R			2700	2600	0.96	150	290	190	0.66	1.31	42	0.29	0.25	6.40	0.26	0.48	26.30	DSS	88.0	0.22
41-R			2700	2600	0.96	150	290	190	0.66	1.31	42	0.42	0.25	6.40	0.26	0.48	26.30	TDS	181.0	0.45
42-R			1800	2600	1.44	150	290	190	0.66	1.31	42	0.58	0.25	6.40	0.26	0.48	26.30	TDS	67.0	0.25
43-R			3600	2600	0.72	150	290	190	0.66	1.31	42	0.58	0.25	6.40	0.26	0.48	26.30	DSS	223.0	0.41
44-R	Petry and Beyer, 2015 [70]	Hollow clay brick	2010	2250	1.12	200	300	190	0.63	1.27	-	1.06	0.50	5.87	0.27	0.94	35.00	TDS	187.0	0.47
45-R			2010	2250	1.12	200	300	190	0.63	1.27	-	1.06	0.50	5.87	0.27	0.94	35.00	TDS	178.0	0.44
46-R			2010	2250	1.12	200	300	190	0.63	1.27	-	1.53	0.50	5.87	0.27	0.94	35.00	TDS	145.0	0.36
47-R			2010	2250	1.12	200	300	190	0.63	1.27	-	0.53	0.50	5.87	0.27	0.94	35.00	TDS	135.0	0.34
48-R			2010	2250	1.12	200	300	190	0.63	1.27	-	0.53	0.50	5.87	0.27	0.94	35.00	TDS	154.0	0.38
49-R	Morandi et al., 2014 [71]	Hollow clay Brick	1350	2140	1.59	350	225	230	1.02	2.04	55	0.15	0.60	6.20	0.49	1.04	10.50	DSS	48.0	0.10
50-R			1350	2140	1.59	350	225	230	1.02	2.04	55	0.45	0.60	6.20	0.49	1.04	10.50	DSS	119.0	0.25
51-R			1350	2140	1.59	350	225	230	1.02	2.04	55	0.65	0.60	6.20	0.49	1.04	10.50	TDS	164.0	0.35
52-R			2700	2140	0.79	350	225	230	1.02	2.04	55	0.45	0.60	6.20	0.49	1.04	10.50	DSS	263.0	0.28
53-R			2700	2140	0.79	350	225	230	1.02	2.04	55	0.65	0.60	6.20	0.49	1.04	10.50	TDS	341.0	0.36
54-R	Martinelli et al., 2016 [72]	Clay brick	1160	1160	1.00	250	250	55	0.22	0.44	0	0.52	0.13	12.31	-	-	30.32	TDS	124.0	0.43
55-R			1160	1160	1.00	250	250	55	0.22	0.44	0	0.52	0.13	12.31	-	-	30.32	TDS	68.0	0.23
56-R			1160	1160	1.00	250	250	55	0.22	0.44	0	0.52	0.13	12.31	-	-	30.32	TDS	104.0	0.36
57-R	Fehling and Stuerz, 2007 [73]	Hollow clay brick	2200	3800	1.73	175	250	240	0.96	1.92	-	1.00	0.28	10.30	-	-	10.30	TDS	160.0	0.42
58-R			2200	3800	1.73	175	250	240	0.96	1.92	-	1.00	0.28	10.30	-	-	10.30	DSS	140.0	0.36
59-R			2200	3800	1.73	175	250	240	0.96	1.92	-	1.00	0.28	10.30	-	-	10.30	TDS	118.0	0.31
60-R			2200	3800	1.73	175	250	240	0.96	1.92	-	1.00	0.28	10.30	-	-	10.30	TDS	147.0	0.38
61-R			2200	3800	1.73	175	250	240	0.96	1.92	-	1.00	0.28	10.30	-	-	10.30	TDS	120.0	0.31
62-R			2200	3800	1.73	175	250	240	0.96	1.92	-	1.00	0.28	10.30	-	-	10.30	TDS	149.0	0.39
63-R			1100	1900	1.73	175	250	240	0.96	1.92	-	1.00	0.28	10.30	-	-	10.30	TDS	60.0	0.31
64-R			1100	1900	1.73	175	250	240	0.96	1.92	-	1.00	0.28	10.30	-	-	10.30	TDS	56.0	0.29
65-R			2200	950	0.43	175	250	240	0.96	1.92	-	0.25	0.28	10.30	-	-	10.30	DSS	72.0	0.19
66-R			2200	3800	1.73	175	250	240	0.96	1.92	-	1.00	0.28	10.30	-	-	10.30	TDS	150.0	0.39
67-R			2200	3800	1.73	175	250	240	0.96	1.92	-	1.00	0.28	10.30	-	-	10.30	TDS	162.0	0.42
68-R			1100	1900	1.73	175	250	240	0.96	1.92	-	1.00	0.28	10.30	-	-	10.30	TDS	70.0	0.36
69-R	Fehling and Stuerz, 2007 [73]	Calcium silicate	1250	2200	1.76	175	175	249	1.42	2.85	-	1.00	0.27	5.93	-	-	24.00	DSS	91.0	0.42
70-R			1250	2200	1.76	175	175	249	1.42	2.85	-	1.00	0.27	5.93	-	-	24.00	TDS	86.0	0.39
71-R	Fehling and Stuerz, 2007 [73]	Lightweight Aerated Concrete	1250	2200	1.76	175	250	240	0.96	1.92	-	1.00	0.25	2.40	-	-	3.31	DSS	90.0	0.41
72-R			1250	2200	1.76	175	250	240	0.96	1.92	-	1.00	0.25	2.40	-	-	3.31	DSS	98.0	0.45
73-R			1250	1100	0.88	175	250	240	0.96	1.92	-	0.50	0.25	2.40	-	-	3.31	DSS	65.0	0.30
74-R			1250	1100	0.88	175	250	240	0.96	1.92	-	0.50	0.25	2.40	-	-	3.31	DSS	61.0	0.28
75-R			1250	2200	1.76	175	250	240	0.96	1.92	-	1.00	0.25	2.40	-	-	3.31	DSS	49.0	0.22
76-R	Magenes et al., 2008 [64]	Lightweight Aerated Concrete	2500	2500	1.00	175	247	240	0.97	1.94	0	0.50	0.25	2.40	-	-	3.31	DSS	125.0	0.29
77-R			2500	2500	1.00	175	247	240	0.97	1.94	0	0.50	0.25	2.40	-	-	3.31	DSS	140.0	0.32
78-R			2500	2500	1.00	175	247	240	0.97	1.94	0	1.00	0.25	2.40	-	-	3.31	DSS	230.0	0.53
79-R			2500	2500	1.00	175	247	240	0.97	1.94	0	1.00	0.25	2.40	-	-	3.31	DSS	230.0	0.53
80-R	Borri et al., 2015 [27]	Clay brick	890	905	1.02	250	240	55	0.23	0.46	0	0.48	0.10	6.00	-	-	20.99	TDS	84.1	0.38
81-R			900	895	0.99	250	240	55	0.23	0.46	0	0.40	0.10	6.00	-	-	20.99	TDS	61.3	0.27
82-R			930	900	0.97	250	240	55	0.23	0.46	0	0.39	0.10	6.00	-	-	20.99	TDS	70.8	0.30
83-R	Marcari et al., 2007 [41]	Tuff stone (‘a sacco’)	1480	1570	1.06	530	-	-	-	-	0	0.50	0.06	1.40	-	-	2.00	TDS	132.0	0.17
84-R	Faella et al., 1992 [74]	Tuff stone (‘a sacco’)	1300	1250	0.96	500	-	-	-	-	0	0.21	0.06	2.00	-	-	3.00	TDS	82.4	0.13
85-R			1300	1250	0.96	500	-	-	-	-	0	0.21	0.06	2.00	-	-	3.00	TDS	83.0	0.13
86-R			1300	1250	0.96	500	-	-	-	-	0	0.52	0.06	2.00	-	-	3.00	TDS	107.7	0.17
87-R			1300	1250	0.96	500	-	-	-	-	0	0.52	0.06	2.00	-	-	3.00	TDS	124.4	0.19
88-R	Faella et al., 1992 [74]	Tuff stone	1300	1250	0.96	500	-	-	-	-	0	0.21	0.12	3.50	-	-	3.50	TDS	102.4	0.16
89-R			1300	1250	0.96	500	-	-	-	-	0	0.52	0.12	3.50	-	-	3.50	TDS	165.7	0.25
90-R	Lourenço et al., 2005 [75]	Dry Stone	1000	1000	1.00	200	200	100	0.50	1.00	0	0.15	0.06	50.00	-	-	82.70	HSS	22.0	0.11
91-R			1000	1000	1.00	200	200	100	0.50	1.00	0	0.15	0.06	50.00	-	-	82.70	HSS	23.0	0.12
92-R			1000	1000	1.00	200	200	100	0.50	1.00	0	0.50	0.07	50.00	-	-	82.70	TDS	42.0	0.21
93-R			1000	1000	1.00	200	200	100	0.50	1.00	0	0.50	0.07	50.00	-	-	82.70	TDS	49.0	0.25

Failure modes: F = Flexural; DSS = Diagonal Sliding Shear; HSS = Horizontal Sliding Shear; TDS = Tensile Diagonal Shear.

**Table 6:** Theoretical results and comparison with the experimental results for regular masonry walls.

Experimental Result			Theoretical Results									Theoretical vs. Experimental Results			
			Flexural Failure (F), *V_th_*_,*F*_				Sliding Failure				Tensile Failure of Units (TDS) *V_th_*_,*TDS*_				
							‘local’ (HSS) *V_th_*_,*HSS*_		‘global’ (DSS) *V_th_*_,*DSS*_						
CASE	V_exp_	Failure Mode	Equation (1)	Equations (2) and (5)	Equation (3)	Equation (4)	Equation (7)	Equation (8)	Equations (9) and (11)	Equation (10)	Equation (12)	*V_th_*_,*min*,*exp*_	*ρ_min_*_,*exp*_	*V_th_*_,*min*_	*ρ_min_*
	(kN)		(kN)	(kN)	(kN)	(kN)	(kN)	(kN)	(kN)	(kN)	(kN)	(kN)	(−)	(kN)	(−)
1-R	75.0	DSS	100.4	98.5	95.8	98.7	83.8	72.3	72.2	76.7	79.5	72.2	0.96	72.2	0.96 (DSS)
2-R	65.0	F	67.7	66.5	64.6	66.7	83.8	72.3	64.9	65.8	71.6	64.6	0.99	64.6	0.99 (F)
3-R	75.0	DSS	104.8	104.0	102.9	104.1	150.9	124.7	79.9	81.2	51.4	79.9	1.07	51.4	***0.69 (TDS)***
4-R	50.0	DSS	53.5	53.3	53.1	53.4	121.4	95.2	61.0	78.0	41.7	61.0	1.22	41.7	***0.83 (TDS)***
5-R	140.0	DSS	200.5	197.3	192.7	197.8	210.0	183.8	117.8	145.3	66.8	117.8	0.84	66.8	***0.48 (TDS)***
6-R	100.0	DSS	104.8	104.0	102.9	104.1	150.9	124.7	79.9	81.2	51.4	79.9	0.80	51.4	***0.51 (TDS)***
7-R	309.0	DSS	455.3	449.1	440.2	450.0	452.7	362.7	383.1	416.9	339.5	383.1	1.24	339.5	***1.10 (TDS)***
8-R	340.0	DSS	455.3	449.1	440.2	450.0	452.7	362.7	383.1	416.9	339.5	383.1	1.13	339.5	***1.00 (TDS)***
9-R	86.0	F	85.4	84.6	83.4	84.7	208.1	163.1	119.5	143.0	110.3	83.4	0.97	83.4	0.97 (F)
10-R	72.0	F	85.4	84.6	83.4	84.7	208.1	163.1	119.5	143.0	110.3	83.4	1.16	83.4	1.16 (F)
11-R	27.7	DSS	28.2	27.6	26.6	27.7	27.9	24.7	17.0	21.4	36.4	17.0	0.61	17.0	0.61 (DSS)
12-R	15.0	DSS	17.1	16.8	16.5	16.9	20.6	17.5	12.0	13.3	32.6	12.0	0.80	12.0	0.80 (DSS)
13-R	119.0	HSS	276.7	272.2	265.8	272.9	83.8	72.4	74.5	77.9	184.9	72.4	0.61	72.4	0.61 (HSS)
14-R	102.0	HSS	172.2	170.5	168.2	170.8	66.3	54.9	56.5	57.3	170.4	54.9	0.54	54.9	0.54 (HSS)
15-R	130.0	TDS	129.5	128.3	126.4	128.4	295.5	235.2	144.5	189.7	99.8	99.8	0.77	99.8	0.77 (TDS)
16-R	166.0	TDS	177.3	174.8	171.3	175.2	329.2	268.8	165.2	232.7	108.7	108.7	0.65	108.7	0.65 (TDS)
17-R	198.0	TDS	244.7	239.6	232.3	240.3	379.8	319.4	196.2	206.4	120.8	120.8	0.61	120.8	0.61 (TDS)
18-R	401.0	TDS	518.1	513.0	505.8	513.8	591.1	470.3	433.4	456.4	299.5	299.5	0.75	299.5	0.75 (TDS)
19-R	500.0	TDS	709.2	699.3	685.0	700.7	658.4	537.7	495.5	537.0	326.1	326.1	0.65	326.1	0.65 (TDS)
20-R	140.6	TDS	228.0	201.9	164.6	205.8	160.4	145.3	112.6	156.0	106.9	106.9	0.76	106.9	0.76 (TDS)
21-R	92.0	TDS	151.0	144.5	135.1	145.4	106.7	91.6	71.0	91.8	84.4	84.4	0.92	71.0	***0.77 (DSS)***
22-R	133.7	TDS	214.5	194.2	165.1	197.2	149.7	134.8	101.9	144.4	75.9	75.9	0.57	75.9	0.57 (TDS)
23-R	90.9	TDS	146.5	140.3	131.5	141.3	105.7	90.8	68.7	91.1	60.4	60.4	0.66	60.4	0.66 (TDS)
24-R	118.0	TDS	190.3	177.2	158.5	179.1	130.2	115.4	87.3	121.3	69.5	69.5	0.59	69.5	0.59 (TDS)
25-R	128.7	TDS	219.1	196.3	163.9	199.8	138.8	127.1	100.7	138.4	87.0	87.0	0.68	87.0	0.68 (TDS)
26-R	84.2	TDS	148.8	142.4	133.1	143.3	93.1	81.4	64.5	84.7	69.1	69.1	0.82	64.5	***0.77 (DSS)***
27-R	141.7	TDS	213.9	194.4	166.6	197.3	169.4	147.6	141.2	178.3	74.2	74.2	0.52	74.2	0.52 (TDS)
28-R	93.9	TDS	158.1	150.7	140.1	151.8	133.8	112.0	107.2	122.4	61.5	61.5	0.66	61.5	0.66 (TDS)
29-R	131.0	TDS	244.1	219.9	185.2	223.5	166.7	149.0	157.1	198.0	136.7	136.7	1.04	136.7	1.04 (TDS)
30-R	91.6	TDS	170.9	163.2	152.3	164.4	120.6	113.0	108.6	125.1	114.4	114.4	1.25	108.6	***1.19 (DSS)***
31-R	213.2	TDS	678.1	632.1	566.3	639.0	429.0	429.0	319.4	319.4	208.1	208.1	0.98	208.1	0.98 (TDS)
32-R	99.1	TDS	225.7	210.4	188.5	212.7	247.5	247.5	153.6	184.3	100.0	100.0	1.01	100.0	1.01 (TDS)
33-R	157.4	TDS	404.2	392.7	376.3	394.5	214.5	214.5	159.7	159.7	166.5	166.5	1.06	159.7	***1.01 (DSS)***
34-R	65.5	DSS	134.5	130.7	125.2	131.3	123.8	123.8	76.8	92.1	80.0	76.8	1.17	76.8	1.17 (DSS)
35-R	91.0	TDS	121.5	119.1	115.7	119.5	151.0	127.6	73.4	85.0	97.4	97.4	1.07	73.4	***0.81 (DSS)***
36-R	103.0	TDS	172.1	166.8	159.1	167.6	185.6	162.2	93.3	107.9	107.7	107.7	1.05	93.3	***0.91 (DSS)***
37-R	131.0	TDS	156.1	153.0	148.6	153.5	170.6	146.4	82.0	92.8	95.8	95.8	0.73	82.0	***0.63 (DSS)***
38-R	148.0	TDS	221.4	214.5	204.7	215.6	213.8	189.6	106.2	119.1	107.9	107.9	0.73	106.2	***0.72 (DSS)***
39-R	141.0	TDS	221.9	218.0	212.4	218.6	260.2	218.1	133.9	146.8	183.0	183.0	1.30	133.9	***0.95 (DSS)***
40-R	88.0	DSS	116.0	115.0	113.7	115.2	203.6	161.5	99.1	103.8	162.4	99.1	1.13	99.1	1.13 (DSS)
41-R	181.0	TDS	165.0	162.9	160.0	163.2	229.0	186.9	114.7	124.3	172.0	172.0	0.95	114.7	***0.63 (DSS)***
42-R	67.0	TDS	98.8	97.1	94.6	97.3	173.6	145.6	61.9	92.6	84.5	84.5	1.26	61.9	***0.92 (DSS)***
43-R	223.0	DSS	394.1	387.2	377.3	388.2	346.8	290.6	178.4	201.5	244.0	178.4	0.80	178.4	0.80 (DSS)
44-R	187.0	TDS	311.1	299.1	281.9	300.9	275.6	253.9	207.1	214.6	232.2	232.2	1.24	207.1	***1.11 (DSS)***
45-R	178.0	TDS	311.1	299.1	281.9	300.9	275.6	253.9	207.1	214.6	232.2	232.2	1.30	207.1	***1.16 (DSS)***
46-R	145.0	TDS	405.6	380.4	344.5	384.2	364.3	342.6	279.4	297.3	256.8	256.8	1.77	256.8	1.77 (TDS)
47-R	135.0	TDS	172.6	169.6	165.3	170.1	175.8	154.1	125.7	146.3	201.0	201.0	1.49	125.7	***0.93 (DSS)***
48-R	154.0	TDS	172.6	169.6	165.3	170.1	175.8	154.1	125.7	146.3	201.0	201.0	1.31	125.7	***0.82 (DSS)***
49-R	48.0	DSS	43.6	43.4	43.2	43.5	198.9	152.6	65.1	73.7	52.4	65.1	1.36	43.2	0.90 (DSS)
50-R	119.0	DSS	124.4	122.7	120.2	122.9	272.6	226.3	96.5	142.5	67.2	96.5	0.81	67.2	***0.56 (TDS)***
51-R	164.0	TDS	173.4	169.9	164.7	170.4	321.8	275.5	117.5	179.0	75.5	75.5	0.46	75.5	0.46 (TDS)
52-R	263.0	DSS	497.6	490.7	480.9	491.7	545.3	452.7	289.6	319.5	201.7	289.6	1.10	201.7	***0.77 (TDS)***
53-R	341.0	TDS	693.7	679.4	658.9	681.6	643.5	550.9	352.5	389.3	226.5	226.5	0.66	226.5	0.66 (TDS)

NOTE: Bold Italics is used to identify the cases in which the predicted failure mechanism does not correspond with the experimental one.

**Table 7:** Mean, standard deviation and CoV associated with the ratios *ρ_min_* and *ρ_min_*_,*exp*_.

Statistical Values	*ρ_min_*_,*exp*_	*ρ_min_*
Mean (−)	0.94	0.82
Stand. Dev. (−)	0.28	0.24
CoV (%)	30.4%	29.4%

**Table 8:** Experimental data on irregular/rubble masonry walls.

Case	Authors	Type of Masonry	*B*	*H*	*λ*	*s*	*σ*_0_	*f_t_*	*f_c_*	*f_bc_*	Failure Mode	*V_exp_*	*τ_av_*
			(mm)	(mm)	(−)	(mm)	(MPa)	(MPa)	(MPa)	(MPa)		(kN)	(MPa)
94-R	Borri et al., 2015 [27]	Double-leaf stone	860	910	1.06	480	0.15	0.05	20.99	36.00	DS	34.3	0.08
95-R			860	900	1.05	480	0.18	0.05	20.99	36.00	DS	37.0	0.09
96-R			900	900	1.00	480	0.18	0.05	20.99	36.00	DS	62.5	0.14
97-IR			880	915	1.04	480	0.31	0.07	20.99	36.00	DS	88.3	0.21
98-IR			930	915	0.98	480	0.29	0.10	20.99	36.00	DS	100.5	0.23
99-IR			880	910	1.03	480	0.12	0.06	20.99	36.00	DS	74.4	0.18
100-IR	Borri et al., 2015 [27]	Double-leaf stone	900	903	1.00	510	0.21	0.04	20.99	36.00	DS	109.3	0.24
101-IR			900	905	1.01	490	0.21	0.04	20.99	36.00	DS	52.0	0.12
102-IR			900	903	1.00	510	0.19	0.04	20.99	36.00	DS	80.7	0.18
103-IR			900	950	1.06	490	0.21	0.04	20.99	36.00	DS	87.6	0.20
104-IR	Vasconcelos and Lourenço, 2009 [10]	Irregular stone	1000	1200	1.20	200	0.88	0.12	18.40	18.40	DS	55.7	0.28
105-IR			1000	1200	1.20	200	1.25	0.12	18.40	18.40	DS	83.0	0.41
106-IR	Vasconcelos and Lourenço, 2009 [10]	Rubble stone	1000	1200	1.20	200	0.88	0.12	18.40	18.40	DS	63.8	0.32
107-IR			1000	1200	1.20	200	1.25	0.12	18.40	18.40	DS	66.0	0.33
108-IR	Magenes et al., 2010 [76]	Double-leaf stone	1250	2500	2.00	320	0.20	0.14	3.28	165.00	DS	48.0	0.12
109-IR			2500	2500	1.00	320	0.50	0.14	3.28	165.00	DS	234.0	0.29
110-IR			2500	2500	1.00	320	0.20	0.14	3.28	165.00	DS	154.0	0.19
111-IR	Silva et al., 2014 [77]	Three-leaf stone	1000	1200	1.20	500	0.50	0.05	2.50	93.40	DS	64.7	0.13
112-IR			1000	1200	1.20	500	0.75	0.05	2.50	93.40	DS	84.5	0.17
113-IR			1000	1200	1.20	500	1.00	0.05	2.50	93.40	DS	93.5	0.19
114-IR	Godio et al., 2019 [26]	Irregular stone	900	900	1.00	200	0.74	0.25	10.34	65.60	DS	76.0	0.42
115-IR			900	900	1.00	200	0.99	0.25	10.34	65.60	DS	90.0	0.50
116-R			900	900	1.00	200	1.52	0.25	10.34	65.60	DS	138.0	0.77
117-R			900	900	1.00	200	1.15	0.25	10.34	65.60	DS	110.0	0.61
118-R			900	900	1.00	200	1.15	0.25	10.34	65.60	DS	108.0	0.60
119-R			900	900	1.00	200	1.15	0.25	10.34	65.60	DS	103.0	0.57
120-R	Gattesco et al., 2015 [78]	Double-leaf stone	1500	2000	1.33	350	0.90	0.13	4.50	7.50	DS	155.0	0.30

**Table 9:** Theoretical results and comparison with the experimental results for irregular/rubble masonries.

Case	*V_exp_*(kN)	Fail. Mode	Flexural Failure (F), *V_th_*_,*F*_ (kN)	Diagonal Shear (DS), *V_th_*_,*DS*_ (kN)			*ρ_F_ = V_th_*_,*F*_*/V_exp_*(−)	*ρ_DS_ = V_th_*_,*DS*_*/V_exp_*(−)		
			Equation (3)	Equations (13) and (16)	Equation (14)	Equation (15)	Equation (3)	Equations (13) and (16)	Equation (14)	Equation (15)
94-R	34.3	DS	56.77	37.0	33.3	37.0	1.66	1.08	0.97	1.08
95-R	37.0	DS	71.67	40.8	36.8	40.8	1.94	1.10	0.99	1.10
96-R	62.5	DS	78.07	44.6	40.2	44.6	1.25	0.71	0.64	0.71
97-IR	88.3	DS	122.50	67.2	60.5	67.2	1.39	0.76	0.68	0.76
98-IR	100.5	DS	127.67	87.8	79.0	89.3	1.27	0.87	0.79	0.89
99-IR	74.4	DS	49.42	42.2	38.0	42.2	0.66	0.57	0.51	0.57
100-IR	109.3	DS	93.86	48.2	43.4	48.2	0.86	0.44	0.40	0.44
101-IR	52.0	DS	89.93	46.2	41.6	46.2	1.73	0.89	0.80	0.89
102-IR	80.7	DS	84.95	46.2	41.6	46.2	1.05	0.57	0.52	0.57
103-IR	87.6	DS	86.08	44.1	39.7	44.1	0.98	0.50	0.45	0.50
104-IR	55.7	DS	135.93	57.6	51.8	57.6	2.44	1.03	0.93	1.03
105-IR	83.0	DS	188.11	67.6	60.8	67.6	2.27	0.81	0.73	0.81
106-IR	63.8	DS	135.93	57.6	51.8	57.6	2.13	0.90	0.81	0.90
107-IR	66.0	DS	188.11	67.6	60.8	67.6	2.85	1.02	0.92	1.02
108-IR	48.0	DS	76.52	57.3	51.6	43.0	1.59	1.19	1.07	0.90
109-IR	234.0	DS	312.89	236.3	212.7	236.3	1.34	1.01	0.91	1.01
110-IR	154.0	DS	196.06	171.9	154.7	171.9	1.27	1.12	1.00	1.12
111-IR	64.7	DS	148.81	69.1	62.2	69.1	2.30	1.07	0.96	1.07
112-IR	84.5	DS	178.57	83.3	75.0	83.3	2.11	0.99	0.89	0.99
113-IR	93.5	DS	178.57	95.5	85.9	95.5	1.91	1.02	0.92	1.02
114-IR	76.0	DS	120.22	89.7	80.8	89.7	1.58	1.18	1.06	1.18
115-IR	90.0	DS	153.68	100.2	90.2	100.2	1.71	1.11	1.00	1.11
116-R	138.0	DS	215.79	119.6	107.7	119.6	1.56	0.87	0.78	0.87
117-R	110.0	DS	174.11	106.5	95.8	106.5	1.58	0.97	0.87	0.97
118-R	108.0	DS	174.11	106.5	95.8	106.5	1.61	0.99	0.89	0.99
119-R	103.0	DS	174.11	106.5	95.8	106.5	1.69	1.03	0.93	1.03
120-R	155.0	DS	253.13	144.1	129.7	144.1	1.63	0.93	0.84	0.93

**Table 10:** Comparison between experimental and theoretical capacities.

Case	*λ* (−)	*V_exp_* (kN)	Failure Mode	Diagonal Shear (DS), *V_th_*_,*DS*_ (kN)			*ρ_DS_ =V_th_*_,*DS*_*/V_exp_*(−)			*ρ_min_ =V_th_*_,*min*_*/V_exp_*(−)
				Equations (13) and (16)	Equation (14)	Equation (15)	Equations (13) and (16)	Equation (14)	Equation (15)	Min
1-R	1.35	75.0	DSS	85.4	76.8	85.4	1.14	1.02	1.14	0.96 (DSS)
3-R	2.00	75.0	DSS	85.4	76.9	64.0	1.14	1.02	0.85	0.69 (**TDS**)
4-R	2.00	50.0	DSS	66.5	59.8	49.9	1.33	1.20	1.00	0.83 (**TDS**)
5-R	2.00	140.0	DSS	114.2	102.8	85.6	0.82	0.73	0.61	0.48 (**TDS**)
6-R	2.00	100.0	DSS	85.4	76.9	64.0	0.85	0.77	0.64	0.51 (**TDS**)
7-R	1.04	309.0	DSS	372.2	334.9	372.2	1.20	1.08	1.20	1.10 (**TDS**)
8-R	1.04	340.0	DSS	372.2	334.9	372.2	1.09	0.99	1.09	1.00 (**TDS**)
11-R	2.45	27.7	DSS	32.7	29.4	20.0	1.18	1.06	0.72	0.61 (DSS)
12-R	2.45	15.0	DSS	26.8	24.1	16.4	1.78	1.61	1.09	0.80 (DSS)
13-R	0.68	119.0	HSS	157.5	141.8	233.4	1.32	1.19	1.96	0.61 (HSS)
14-R	0.68	102.0	HSS	133.5	120.2	197.8	1.31	1.18	1.94	0.54 (HSS)
15-R	1.60	130.0	TDS	178.2	160.3	167.0	1.37	1.23	1.28	0.77 (TDS)
16-R	1.60	166.0	TDS	196.8	177.1	184.5	1.19	1.07	1.11	0.65 (TDS)
17-R	1.60	198.0	TDS	221.8	199.6	207.9	1.12	1.01	1.05	0.61 (TDS)
18-R	0.80	401.0	TDS	534.5	481.0	668.1	1.33	1.20	1.67	0.75 (TDS)
19-R	0.80	500.0	TDS	590.3	531.3	737.9	1.18	1.06	1.48	0.65 (TDS)
20-R	1.43	140.6	TDS	137.7	123.9	137.7	0.98	0.88	0.98	0.76 (TDS)
21-R	1.43	92.0	TDS	102.4	92.2	102.4	1.11	1.00	1.11	0.77 (**DSS**)
22-R	1.48	133.7	TDS	132.0	118.8	132.0	0.99	0.89	0.99	0.57 (TDS)
23-R	1.48	90.9	TDS	102.7	92.4	102.7	1.13	1.02	1.13	0.66 (TDS)
24-R	1.48	118.0	TDS	119.9	107.9	119.9	1.02	0.91	1.02	0.59 (TDS)
25-R	1.41	128.7	TDS	127.4	114.7	127.4	0.99	0.89	0.99	0.68 (TDS)
26-R	1.41	84.2	TDS	97.3	87.5	97.3	1.16	1.04	1.16	0.77 (**DSS**)
27-R	1.43	141.7	TDS	139.9	125.9	139.9	0.99	0.89	0.99	0.52 (TDS)
28-R	1.43	93.9	TDS	114.6	103.1	114.6	1.22	1.10	1.22	0.66 (TDS)
29-R	1.37	131.0	TDS	138.2	124.4	138.2	1.05	0.95	1.05	1.04 (TDS)
30-R	1.37	91.6	TDS	107.7	96.9	107.7	1.18	1.06	1.18	1.19 (**DSS**)
31-R	0.69	213.2	TDS	215.6	194.0	311.4	1.01	0.91	1.46	0.98 (TDS)
32-R	1.20	99.1	TDS	103.6	93.3	103.6	1.05	0.94	1.05	1.01 (TDS)
33-R	0.69	157.4	TDS	159.2	143.3	230.0	1.01	0.91	1.46	1.01 (**DSS**)
34-R	1.20	65.5	DSS	76.5	68.9	76.5	1.17	1.05	1.17	1.17 (DSS)
35-R	1.07	91.0	TDS	99.5	89.5	99.5	1.09	0.98	1.09	0.81 (**DSS**)
36-R	1.07	103.0	TDS	116.0	104.4	116.0	1.13	1.01	1.13	0.91 (**DSS**)
37-R	1.03	131.0	TDS	116.6	104.9	116.6	0.89	0.80	0.89	0.63 (**DSS**)
38-R	1.03	148.0	TDS	136.7	123.0	136.7	0.92	0.83	0.92	0.72 (**DSS**)
39-R	0.96	141.0	TDS	184.5	166.1	191.6	1.31	1.18	1.36	0.95 (**DSS**)
40-R	0.96	88.0	DSS	148.7	133.8	154.4	1.69	1.52	1.75	1.13 (DSS)
41-R	0.96	181.0	TDS	165.7	149.2	172.1	0.92	0.82	0.95	0.63 (**DSS**)
42-R	1.44	67.0	TDS	85.2	76.7	85.2	1.27	1.14	1.27	0.92 (**DSS**)
43-R	0.72	223.0	DSS	245.9	221.3	340.5	1.10	0.99	1.53	0.80 (DSS)
44-R	1.12	187.0	TDS	209.0	188.1	209.0	1.12	1.01	1.12	1.11 (**DSS**)
45-R	1.12	178.0	TDS	209.0	188.1	209.0	1.17	1.06	1.17	1.16 (**DSS**)
46-R	1.12	145.0	TDS	209.0	188.1	209.0	1.44	1.30	1.44	1.77 (TDS)
47-R	1.12	135.0	TDS	221.7	199.5	221.7	1.64	1.48	1.64	0.93 (**DSS**)
48-R	1.12	154.0	TDS	195.5	176.0	195.5	1.27	1.14	1.27	0.82 (**DSS**)
49-R	1.59	48.0	DSS	99.6	89.7	94.3	2.08	1.87	1.96	0.90 (DSS)
50-R	1.59	119.0	DSS	131.8	118.6	124.7	1.11	1.00	1.05	0.56 (**TDS**)
51-R	1.59	164.0	TDS	149.4	134.5	141.4	0.91	0.82	0.86	0.46 (TDS)
52-R	0.79	263.0	DSS	395.3	355.8	498.8	1.50	1.35	1.90	0.77 (**TDS**)
53-R	0.79	341.0	TDS	448.3	403.4	565.6	1.31	1.18	1.66	0.66 (TDS)
54-R	1.00	124.0	DDS	83.7	75.4	83.7	0.68	0.61	0.68	-
55-R	1.00	68.0	DDS	83.7	75.4	83.7	1.23	1.11	1.23	-
56-R	1.00	104.0	DDS	83.7	75.4	83.7	0.81	0.72	0.81	-
57-R	1.73	160.0	TDS	153.7	138.3	133.4	0.96	0.86	0.83	-
58-R	1.73	140.0	DSS	153.7	138.3	133.4	1.10	0.99	0.95	-
59-R	1.73	118.0	TDS	153.7	138.3	133.4	1.30	1.17	1.13	-
60-R	1.73	147.0	TDS	153.7	138.3	133.4	1.05	0.94	0.91	-
61-R	1.73	120.0	TDS	153.7	138.3	133.4	1.28	1.15	1.11	-
62-R	1.73	149.0	TDS	153.7	138.3	133.4	1.03	0.93	0.90	-
63-R	1.73	60.0	TDS	76.8	69.1	66.7	1.28	1.15	1.11	-
64-R	1.73	56.0	TDS	76.8	69.1	66.7	1.37	1.23	1.19	-
65-R	0.43	72.0	DSS	148.3	133.5	171.7	2.06	1.85	2.39	-
66-R	1.73	150.0	TDS	153.7	138.3	133.4	1.02	0.92	0.89	-
67-R	1.73	162.0	TDS	153.7	138.3	133.4	0.95	0.85	0.82	-
68-R	1.73	70.0	TDS	76.8	69.1	66.7	1.10	0.99	0.95	-
69-R	1.76	91.0	DSS	85.4	76.9	72.8	0.94	0.84	0.80	-
70-R	1.76	86.0	TDS	85.4	76.9	72.8	0.99	0.89	0.85	-
71-R	1.76	90.0	DSS	81.5	73.4	69.5	0.91	0.82	0.77	-
72-R	1.76	98.0	DSS	81.5	73.4	69.5	0.83	0.75	0.71	-
73-R	0.88	65.0	DSS	94.7	85.2	107.6	1.46	1.31	1.66	-
74-R	0.88	61.0	DSS	94.7	85.2	107.6	1.55	1.40	1.76	-
75-R	1.76	49.0	DSS	81.5	73.4	69.5	1.66	1.50	1.42	-
76-R	1.00	125.0	DSS	189.4	170.5	189.4	1.52	1.36	1.52	-
77-R	1.00	140.0	DSS	189.4	170.5	189.4	1.35	1.22	1.35	-
78-R	1.00	230.0	DSS	244.6	220.1	244.6	1.06	0.96	1.06	-
79-R	1.00	230.0	DSS	244.6	220.1	244.6	1.06	0.96	1.06	-
80-R	1.02	84.1	TDS	53.9	48.5	53.9	0.64	0.58	0.64	-
81-R	0.99	61.3	TDS	51.2	46.1	51.5	0.84	0.75	0.84	-
82-R	0.97	70.8	TDS	52.3	47.1	54.1	0.74	0.67	0.76	-
83-R	1.06	132.0	TDS	139.3	125.3	139.3	1.05	0.95	1.05	-
84-R	0.96	82.4	TDS	84.9	76.4	88.3	1.03	0.93	1.07	-
85-R	0.96	83.0	TDS	84.9	76.4	88.3	1.02	0.92	1.06	-
86-R	0.96	107.7	TDS	124.6	112.1	129.6	1.16	1.04	1.20	-
87-R	0.96	124.4	TDS	124.6	112.1	129.6	1.00	0.90	1.04	-
88-R	0.96	102.4	TDS	129.0	116.1	134.1	1.26	1.13	1.31	-
89-R	0.96	165.7	TDS	180.1	162.1	187.3	1.09	0.98	1.13	-
90-R	1.00	22.0	HSS	22.4	20.2	22.4	1.02	0.92	1.02	-
91-R	1.00	23.0	HSS	22.4	20.2	22.4	0.98	0.88	0.98	-
92-R	1.00	42.0	TDS	39.9	36.0	39.9	0.95	0.86	0.95	-
93-R	1.00	49.0	TDS	39.9	36.0	39.9	0.82	0.73	0.82	-

NOTE: Bold is used to identify the cases in which the predicted failure mechanism does not correspond with the experimental one.

**Table 11:** Mean, standard deviation and CoV associated with the ratio *ρ_DS_*.

Slenderness	Statistical Values	*ρ_DS_* Equations (13) and (16)	*ρ_DS_* Equation (14)	*ρ_DS_* Equation (15)
*λ* < 1 (23 data)	Mean (−)	1.23	1.10	1.45
	Stand. Dev. (−)	0.30	0.27	0.41
	CoV (%)	24.2%	24.2%	28.1%
1 ≤ *λ* ≤ 1.5 (39 data)	Mean (−)	1.09	0.98	1.09
	Stand. Dev. (−)	0.20	0.18	0.20
	CoV (%)	18.3%	18.3%	18.3%
*λ* > 1.5 (28 data)	Mean (−)	1.17	1.05	0.99
	Stand. Dev. (−)	0.29	0.26	0.27
	CoV (%)	25.1%	25.1%	27.5%
all tests (90 data)	Mean (−)	1.15	1.03	1.15
	Stand. Dev. (−)	0.26	0.23	0.34
	CoV (%)	22.7%	22.7%	29.3%

**Table 12:** Mean, standard deviation and CoV associated with the ratio *ρ*.

Slenderness	Statistical Values	*ρ_DS_* Equations (13) and (16)*	*ρ_DS_* Equation (14)*
*λ* < 1 (23 data)	Mean (−)	0.82	0.74
	Stand. Dev. (−)	0.20	0.18
	CoV (%)	24.2%	24.2%
1 ≤ *λ* ≤ 1.5 (39 data)	Mean (−)	0.86	0.77
	Stand. Dev. (−)	0.21	0.19
	CoV (%)	24.0%	24.0%
*λ* > 1.5 (28 data)	Mean (−)	1.17	1.05
	Stand. Dev. (−)	0.29	0.26
	CoV (%)	25.1%	25.1%
all tests (90 data)	Mean (−)	0.94	0.85
	Stand. Dev. (−)	0.28	0.25
	CoV (%)	29.3%	29.3%

## Data Availability

Not applicable.
